# Can scent-detection dogs detect the stress associated with trauma cue exposure in people with trauma histories? A proof-of-concept study

**DOI:** 10.3389/falgy.2024.1352840

**Published:** 2024-03-28

**Authors:** Laura Kiiroja, Sherry H. Stewart, Simon Gadbois

**Affiliations:** ^1^ Canine Olfaction Lab, Department of Psychology and Neuroscience, Dalhousie University, Halifax, NS, Canada; ^2^ Mood, Anxiety, and Addictions Comorbidity (MAAC) Lab, Department of Psychiatry, Dalhousie University, Halifax, NS, Canada

**Keywords:** odour detection, scent-detection canines, biomedical alert dogs, psychiatric service dogs, trauma, PTSD

## Abstract

**Introduction:**

Post-traumatic stress disorder (PTSD) is an impairing mental health condition with high prevalence among military and general populations alike. PTSD service dogs are a complementary and alternative intervention needing scientific validation. We investigated whether dogs can detect putative stress-related volatile organic compounds (VOCs) in the breath of people with trauma histories (54% with PTSD) exposed to personalized trauma cues.

**Methods:**

Breath samples were collected from 26 humans over 40 experimental sessions during a calm (control breath sample) and stressed state induced by trauma cue exposure (target breath sample). Two scent detection canines were presented with the samples in a two alternative forced choice (2AFC) discrimination and yes/no detection task. The 2AFC task assessed the dogs' ability to discriminate between the two states within the breath samples of one individual. The detection task determined their ability to generalize the target odour across different individuals and different stressful events of one individual. Signal Detection Theory was applied to assess dogs' sensitivity, specificity, precision, and response bias.

**Results:**

The dogs performed at ∼90% accuracy across all sample sets in the discrimination experiment, and at 74% and 81% accuracy, respectively, in the detection experiment. Further analysis of dog olfactory performance in relation to human donor self-reported emotional responses to trauma cue exposure suggested the dogs may have been detecting distinct endocrine stress markers. One dog's performance correlated with the human donors' self-reported fear responses and the other dog's performance correlated with the human donors' self-reported shame responses. Based on these correlations between dog performance and donor self-report measures, we speculate that the VOCs each dog was detecting likely originated from the sympathetico-adreno-medullary axis (SAM; adrenaline, noradrenaline) in the case of the first dog and the hypothalamo-pituitary-adrenal axis (HPA; glucocorticoids) in the case of the second dog.

**Conclusion:**

Our proof-of-concept study is the first to demonstrate that some dogs can detect putative VOCs emitted by people with trauma histories when experiencing distress theoretically associated with the intrusion and arousal/reactivity symptoms of PTSD. Results have potential to improve the effectiveness and training protocol of PTSD service dogs with a focus on enhancing their alert function.

## 1 Introduction

Post-traumatic stress disorder (PTSD) is a trauma- and stress-related disorder in the *Diagnostic and Statistical Manual of Mental Disorders, 5th edition* [DSM-5; (1)] involving a persistent stress response to experiencing/witnessing a life-threatening or catastrophic event (2, 3) such as combat, sexual/physical assault, or disaster (1). PTSD symptoms fall into four clusters: intrusion/re-experiencing (e.g., flashbacks, nightmares), hyperarousal (e.g., hypervigilance, sleep perturbations), avoidance (avoiding trauma reminders), and cognition/mood symptoms (e.g., emotional numbing, negative emotions) (1, 2). PTSD is often comorbid with other psychiatric disorders (e.g., mood, anxiety, and substance use) (1, 2, 4–6) and physical health conditions (e.g., endocrinological, cardiovascular, and pain) (2, 7). Associated features include suicidal ideation (8) and impairments to family/relationships (9, 10), work (11, 12), well-being, and quality of life (13, 14).

PTSD is prevalent [7.8% lifetime prevalence in the U.S. general population (15), 9.2% in Canada (16)], particularly among veterans (17, 18) [up to 23%–30% (18, 19)]. Many more trauma-exposed individuals experience subthreshold PTSD symptoms. Developing effective treatments for full and subthreshold PTSD is paramount.

One complementary/alternative treatment for PTSD involves psychiatric service dogs–assistance dogs permanently placed with a patient and trained to help them (20). Trained tasks include alerting to early signs of intrusion/hyperarousal symptoms and interrupting/diffusing these episodes (20–25). Growing evidence links service dogs use with clinically-significant long-term decreases in PTSD symptomatology, with the strongest effects for intrusion/hyperarousal symptoms (20–26). PTSD service dogs are associated with increased quality of life and improved family and social functioning/integration (22–24).

Interrupting or alerting to episodes of anxiety/distress (e.g., flashbacks, nightmares) is reported as within the top three most appreciated and frequently-used trained tasks by veterans with a PTSD service dog (24, 27). Most service dog providers consider dogs' ability to interrupt/alert to such episodes a task requiring training (3, 21, 24, 28–30). Currently, PTSD service dogs are trained to respond to physical signs (e.g., fidgeting, fist-clenching) of upcoming intrusion/hyperarousal symptoms (e.g., flashbacks, anger) (3, 24, 28). We investigated whether dogs can detect the early onset of these episodes via the breath of people with trauma histories when exposed to trauma reminders. If reliance on olfactory cues is possible, service dogs might be trained to alert to upcoming intrusion/hyperarousal symptoms before physical signs manifest (31) and prior to patient awareness (7). Early distraction could remind patients to use skills learned in psychotherapy [e.g., mindfulness, relaxation (28)], increasing these skills' efficacy and preventing symptom escalation (30).

## 2 Background information

### 2.1 The basics of using scent-detection dogs in medicine

Canines' sense of smell is 10,000–100,000 times more sensitive than humans' (32). Research is investigating opportunities to apply dogs' acute olfaction to biomedical detection and alert tasks like detecting human cancers (33–38), viruses [e.g., COVID-19; (39)], or parasites [e.g., malaria; (40)], and alerting to hypoglycemia (41), seizures (42), and dangerous bacteria (43, 44).

Three categories of individual human odours are: the stable “primary odour” based on the individual's genetics, age, and sex; the changing “secondary odour” based on endogenous dietary and environmental factors, including pathological status; and the “tertiary odour” based on exogenous factors like personal hygiene products (45, 46). These elements determine the individual scent profile of volatile organic compounds (VOCs) emanated from the human body (e.g., isoprene, monoterpenes) (45). As VOC molecules evaporate/sublimate, dogs can detect them from samples including breath, urine, and sweat (46, 47); greatest success has been achieved with breath (48).

Certain medical conditions alter the VOCs cells release into the respiratory system (41, 45), resulting in the condition's “signature scent” (48) or less specific olfactory biomarkers dogs could be trained to detect. Although it is unclear if every condition has its own VOC pattern, disease-specific profiles have been identified in several diseases/infections/metabolic changes in humans (e.g., cancers, cystic fibrosis, diabetes) (45, 47, 49, 50).

### 2.2 Evidence of the canine potential to detect human stress-related VOCs

There is preliminary evidence of dogs’ ability to detect VOCs associated with elevated human stress levels. One study (51) presented 31 pet dogs with salivary, interdigital, and perianal secretion samples from three dog donors and sweat samples from four human donors during one joyful, one neutral, and two stressful situations (i.e., a fear-inducing video and running for the humans). Regardless of donor species, dogs displayed higher cardiac activity and behavioural alertness and anxiety when sniffing samples collected during stressful vs. neutral/joyful situations (51).

Another study (52) collected sweat samples from eight humans after watching a fear- or joy-inducing video. Forty pet dogs displayed more stress-related behaviours and arousal (e.g., elevated heart rate) when sniffing pooled samples from the fear vs. joy or control (no human odour) conditions (52). A third study (53) collected breath and sweat samples from 36 humans immediately before and after a mental arithmetic task and validated their stress by blood pressure, heart rate, and self-report. The validated samples were then presented to four formerly-trained scent-detection dogs in a three alternative forced choice discrimination task (baseline and stress samples of the same donor, and a blank sample). Dogs were able to discriminate between stress and neutral samples by signalling the stress sample with an average accuracy of 93.75%.

The above-described studies suggest human stress responses involve VOCs that at least some dogs can detect, although the specific stress biomarker(s) dogs rely on remain unclear. Technological advancements have also successfully detected human stress response biomarkers in breath (54, 55) and a variety of bodily fluids [e.g., urine, blood, saliva (56)]. One pilot study (55) collected breath samples from 22 donors during a stress-inducing arithmetic test and a neutral (classical music) condition. Gas chromatography-mass spectrometry analysis detected six stress compounds with indole and 2-methyl-pentadecane the most discriminant. Their model demonstrated 83.3% sensitivity and 91.6% specificity (female donor samples), and 100% sensitivity and 90% specificity (male donor samples).

Another study (54) recruited 14 donors and subjected them to a stressful arithmetic task with a less and more intense version and a control condition (relaxing videos). Breath profiling by gas chromatography/ion mobility spectrometry revealed six stress-sensitive compounds with benzaldehyde common to the previously discussed study (55). The model demonstrated 78.5% sensitivity and 71.5% specificity (more intense stressor), and 61.5% sensitivity and 71.4% specificity (less intense stressor) (54). There is currently no technological sensor able to detect all stress biomarkers or simultaneously detect multiple biomarkers (regardless of VOC source) in a way allowing quick, reliable monitoring of an individual's stress levels (56).

Dogs might have an advantage as they likely possess a “sensor” capable of a more comprehensive and less specialised perception of the array of stress volatiles. There are several reasons why dogs could be sensitive to human stress VOCs. First, dogs might have developed olfactory recognition of human emotions during domestication (57). Alternatively, from an ethological viewpoint, it would be advantageous for predator species to be able to perceive volatiles indicating distress (and thus vulnerability/weakness) in prey (58).

Another possible mechanism is emotional contagion, which occurs when “another's emotional state triggers a similar emotional response in an observer” (59, p. 852). Indeed, dogs exhibit behaviours indicative of emotional contagion in response to visual behavioural signs of human distress (e.g., crying) (59–61). Synchronisation is a broader term, defined as “doing the same thing, at the same time and at the same place, as others” (62, p. 181). Synchronisation has several adaptive values from cooperative defence against predators to raising offspring (62). Dogs have been shown to synchronise with their guardians when encountering unfamiliar objects/people [e.g., (63, 64)]. Synchronisation does not necessarily involve emotional contagion. Although studies of both emotional contagion and synchronisation thus far have involved dogs' responses to visual and audible behavioural cues in humans, certain VOCs may also contribute to eliciting these mechanisms in dogs.

### 2.3 The endocrinology of human stress response associated with PTSD

The main endocrinological factor characterising PTSD/anxiety disorders is chronic amygdalar and stress response over-activity (7, 65, 66). The amygdala is a part of the limbic system responsible for processing fear and other negative emotions, including both innate and conditioned aversive stimuli. Sensory information first reaches the amygdala that then activates the autonomic nervous system, after which the information is sent to the frontal/temporal lobes for further processing (7, 66). As amygdalar processing is often unconscious, informing the prefrontal cortex is not required before experiencing anxiety/fear. People may exhibit physiological stress/fear responses before being consciously aware of the stressor (7).

For PTSD, anxiety, and depression, there is an imbalance between amygdalar activity and prefrontal cortical activity–the amygdala is markedly over-active and the activation of the medial prefrontal cortex is to some extent impaired (2, 66). Without cortical processing, an aroused amygdala stimulates the sympathetic nervous system^1^, which proceeds to secrete stress-response hormones (7, 65). Two endocrine subsystems play a major role in re-establishing homeostasis in response to a stressor: the sympathetic-adreno-medullar (SAM) and the hypothalamic-pituitary-adrenal (HPA) axes. The SAM axis involves the catecholamines adrenaline and noradrenaline. The HPA axis involves the glucocorticoids cortisol and corticosterone (7, 65, 67).

The SAM axis reacts instantaneously: within milliseconds of an exposure to a stressor, the sympathetic nervous system engages the adrenal medulla to produce adrenaline and noradrenaline (7, 65, 67, 68). Reactions through the HPA axis unfold more slowly: the adrenal cortices start releasing glucocorticoids within minutes or hours from exposure (7, 65, 67). The endocrine sequence is also longer: the HPA axis requires the hypothalamus to signal the pituitary gland (via corticotropin releasing hormone) which must activate the adrenal cortex (via adrenocorticotropic hormone) (31).

The function of the SAM axis response is to prepare the organism for a sudden increase in energy demands, i.e., immediate fight-or-flight. Oxygen intake is increased by elevating respiration rate; heart rate is elevated; blood flow to the muscles and blood glucose levels are boosted to prepare for movement; processes not immediately necessary for survival (e.g., digestion, reproduction, growth) are inhibited; pain perception is blunted; and alertness and sensory/learning/memory functions are enhanced (7, 65, 67). The HPA axis reactions (glucocorticoids) support these processes in the long term. They are involved in mediating the stress reaction, recovering from it, and preparing for the next possible stressor(s) (7).

### 2.4 Goals and hypotheses

We investigated whether dogs could be trained to alert to the early onset of PTSD intrusion/hyperarousal symptoms by relying on olfactory cues. The goal was to determine whether dogs can detect and discriminate between breath samples of donors with a trauma exposure history and varying levels of PTSD symptoms, collected immediately before and during exposure to cues related to their personal traumatic experiences. This is the first study to investigate canine ability to detect stress volatiles theoretically involved in PTSD intrusion/hyperarousal symptoms.

We proposed three hypotheses. First, we hypothesised that at least some dogs can discriminate between breath samples collected from the same human donors during a personalised trauma cue vs. resting baseline. This requires that, when presented with the two samples, the canines can detect VOCs associated with stress-induced PTSD intrusion/arousal symptoms.

Second, we hypothesised that at least some dogs can detect the trauma cue breath samples across different individuals (or the same individual in different contexts) when presented with one sample (baseline or trauma cue) at a time. In real-life, dogs would not have simultaneous breath samples to compare but would be presented with one “sample”. Confirmation of this second hypothesis would provide considerably stronger evidence for training PTSD alert dogs.

Finally, we hypothesised a positive correlation between dogs' performance and donors' self-reported negative affect during the trauma cue exposure (indices of human distress when confronting trauma reminders).

## 3 Methods

Ethical approvals were obtained from the Dalhousie University Committee on Laboratory Animals and Nova Scotia Health Authority Research Ethics Board.

### 3.1 Human donors

Human donors were recruited from a study on neurocognitive mechanisms underlying trauma–cannabis use links (69, 70). Donors provided informed consent, including agreement to donate breath samples for this study. Donors had to be aged 19–65, have no severe mental illness (bipolar disorder, psychosis), report on the Life Events Checklist (LEC) (71) having experienced 1+ Criterion A trauma of a DSM-5 (1) PTSD diagnosis, and using 1+ g of cannabis/week over the last month; cannabis use was not a focus of this canine olfaction study.

Breath samples were collected from 26 donors aged 20–53 years (mean age = 31.2). Eight were male, 18 female. The self-report PTSD Checklist (PCL-5) (72, 73) assessed PTSD symptom severity (possible range = 0–80) and estimated the proportion with a likely PTSD diagnosis. The Clinician-Administered PTSD Scale for DSM-5 (CAPS-5) (74), a clinical interview, diagnosed PTSD and provided a second PTSD symptom severity measure (CAPS-5 symptom count; possible range = 0–20) (75).

The mean PCL-5 score was 45.08 (median = 44, range = 25–73)—above the cut-point of 38 for a likely PTSD diagnosis (76). Eighteen (69.2%) scored above this cut-point. The CAPS-5 interview confirmed a PTSD diagnosis in 14 (53.8%). The mean CAPS-5 symptom count was 10.85 (median = 10.50, range = 2–19); thus, the average donor acknowledged ∼11 PTSD symptoms.

To assess for cannabis' possible influence on dog performance, the self-report Cannabis Use Disorder Identification Test-Revised (CUDIT-R) (77) evaluated CUD symptom severity. On a 0–32 scale, the mean was 11.35 (median = 12, range = 3–20)–above the cut-point of 8 for hazardous cannabis use (77). Fourteen scored above the cut-point of 12 (77) for a likely CUD diagnosis (53.8%).

### 3.2 Canine participants

Initially, 25 pet dogs of different breeds were enrolled and commenced scent-detection training at Dalhousie's Canine Olfaction (CO) Lab. Dogs were recruited through personal contacts, e-mails to guardians who had expressed interest in volunteering their dogs, and word-of-mouth. Four dogs who passed the training criteria (Supplementary Material S1) and demonstrated outstanding motivation, stamina, and work drive were selected for training with the target odour. Two (Ivy and Callie) reached sufficiently accurate and consistent performance levels to suggest they had learned to identify the target odour and were ready for testing with novel donor samples (Section 3.5.1).

Ivy is a spayed female working-line Red Golden Retriever who was 5–6 years old during this study. Callie is a spayed female mix of German Shepherd and Belgian Malinois who was 3–4 years old during this study. Callie's parents came from a working-line pedigree but were not active working dogs themselves. Both Ivy and Callie are companion dogs who live with their guardians and were brought to the CO Lab, one dog at a time, 1–3 day(s)/week. Callie had no experience in scent-detection work prior to this study. Ivy had participated in other CO Lab studies since she was 2 years old: discriminating ketogenic from normal cow breath and searching for endangered wood turtles. She had not previously worked with human breath samples.

The small number of dogs does not pose a limitation to this study: the goal was not to demonstrate that all dogs can detect the stress VOCs emanating from people with trauma histories in response to trauma cues but to provide evidence that some dogs can, at high accuracy. An increased number of dogs would not meaningfully contribute to testing this study's hypotheses. High attrition rates based on selection criteria and performance are typical in scent-dog training (78); this research is based on the few dogs to the far-right of the normal distribution, i.e., experts.

### 3.3 Samples

The parent study involved two stress-induction experimental sessions with the donors–the interview session (70, 79) and the imaging session (69)–both at the Biomedical Translational Imaging Centre (BIOTIC), Queen Elizabeth II Health Sciences Centre, Halifax, Canada. At the end of each session, the donor was provided financial compensation: $65CAD (interview session); $150CAD (imaging session).

**Figure 1:**
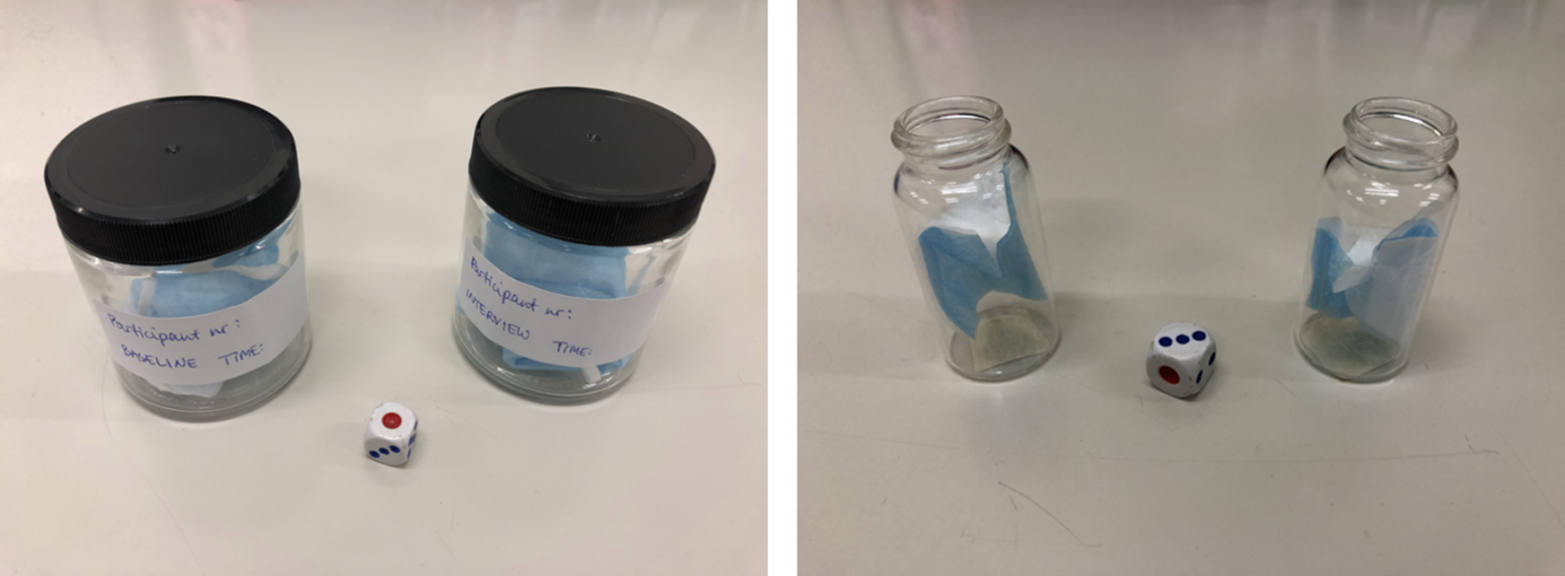
Face masks in the glass sample collection jars (left) and pieces of the masks in the glass vials (right).

In each session, two breath samples were collected via disposable medical-grade face masks. The experimenter was trained in sample collection procedures, wore a medical grade mask themselves, and used a new pair of disposable powder-free latex gloves while handling each mask. The experimenter recorded the duration each mask was worn, and this variable was used in subsequent analyses. After collection, masks were stored in separate marked 4 oz glass jars with plastic caps (Figure 1) and sealed together in a marked Ziploc bag as one sample set (i.e., baseline and trauma cue samples of each donor's session). The bag was placed in a cooler containing an ice pack and transferred immediately to the CO Lab where it was stored in a 4°C fridge.

All 26 humans participated in the interview session. A first mask (resting baseline) was supplied and worn at the session's start, while providing informed consent and completing questionnaires (70, 79). An identical second mask was worn during the interview portion. Interview methods used in an earlier project by the same team were employed (80): three semi-structured interviews were conducted as a cue exposure (79). Interviews included one about a personal neutral event (e.g., grocery shopping), another about a personal event involving the donor's cannabis use, and a third about their personal trauma experience. The trauma and neutral interviews were conducted first in a randomised order followed by the cannabis interview. Donors with more than one lifetime trauma focused on the most distressing. The second mask functioned as the trauma/stress sample as the trauma interview was designed to induce distress. To simplify procedures in the parent study (70, 79), donors wore a single mask during all three interviews since the mask would contain stress volatiles emanated during the trauma interview regardless of it having also been worn during the other two interviews.

Breath samples were collected from 14 imaging sessions (from 13 donors^2^). The parent study's additional eligibility criteria for the imaging session included being right-handed, not having medical contraindications to scanning, and willingness to participate in imaging (69). A mask provided by hospital staff at the entrance and worn until entering the scanner functioned as the baseline breath sample. The experimenter handed the donor another face mask to wear during scanning. That second mask functioned as the trauma/stress sample as the donor was presented with audio-visual and textual cues related to their personal trauma (derived from the interview session). As in the interview session, donors were also exposed to personally-relevant neutral and cannabis cues (derived from the interview session) while wearing the trauma/stress mask. The three cues (trauma, neutral, cannabis) were displayed in random order through headphones and on the screen visible within the scanner.

Unlike in the interview session, the baseline and trauma masks of the imaging sessions were not identical. All were medical-grade disposable masks of light blue colour and visually identical; however, the baseline mask for the imaging session was provided by the hospital and could have been manufactured by another company than the masks provided by our team during scanning. In addition, a single experimenter removed the metal wires from all imaging masks (for safety reasons during scanning), wearing latex gloves and a medical-grade mask. Dogs were only presented with fabric mask pieces (never the nose wire or ear loops). In every other respect, the sample collection/handling/storage procedures for the imaging session were identical to the interview session.

For both sessions, donors were instructed to: stay abstinent from alcohol/drugs for 12 h and caffeine for 2 h prior to the sessions (verified via self-reports and urine tests); not eat/drink anything but still water during each session; not wear make-up, perfume, or scented products on their face; and refrain from touching the mask. Donors mouth-rinsed with still water before donning the interview session baseline mask (mouth-rinsing at the hospital entrance was not practical for the imaging session). To minimise sample contamination, donors donned and removed masks by touching only the elastic ear loops.

In total, 40 breath sample sets from 26 individuals were collected. Of these 26, 13 donated one set (from the interview session), 12 donated two (from both sessions on non-consecutive days), and one donated three (one interview and two imaging session sets, all on separate days). The interviews allowed collection of individualised information to create standardised cues for the imaging session (69). Although all were encouraged to attend both sessions, agreeing to the imaging session was not required to participate in the interview session study.

Samples were prepared for presentation to the dogs in the CO Lab by LK, wearing powder-free latex gloves and a medical-grade mask. The sample preparation began 15–30 min prior to dog testing. LK first disinfected the jars. Having changed gloves, she then procured two sterilised 20 ml glass vials (Figure 1). Small pieces of masking tape were attached to the bottom of each glass vial. The donor number, human session number, and odour condition (“S−” for baseline; “S+” for trauma cue) were written in pencil on the tape and sterilised plastic caps.

After another glove change, LK removed/unfolded the baseline face mask from the jar and cut a small piece of fabric using sterilised scissors. The piece was inserted in the glass vial marked with “S−” using sterilised metal tweezers. She then returned the rest of the mask to the jar, changed gloves again, capped the vial and jar, and repeated the procedure with the trauma cue mask using a different set of sterilised scissors/tweezers. The two vials were sealed together in a Ziploc bag until testing began. The jars containing the masks were preserved in the fridge within their original bag immediately after sample preparation, for future use.

### 3.4 Sample collection outcome measures

As no gold standard is currently available to determine whether a breath sample contains sufficient stress volatiles to confirm its “stressed” status, one can only indirectly assess the sample's validity by measuring the donor's physiological (e.g., heart rate, blood pressure, stress hormones), behavioural, or psychological stress indicators during sample collection. We measured the latter. During the interview session, the well-validated (81) Positive and Negative Affect Schedule (PANAS) (82) assessed donors' state affect at baseline and during each cue exposure (trauma, neutral, cannabis). For each item (10 positive, 10 negative affect), the donor indicated how they felt at baseline or during the cue exposure using a 6-point scale (0 = very slightly or not at all; 5 = extremely). The donor's state negative affect was calculated by summing the 10 negative affect items (e.g., “distressed”, “irritable”, “ashamed”, “nervous”; *α* = 0.770). We also created a State Anxiety composite from the PANAS (sum of 6 negative affect items selected by experts to reflect anxiety: “distressed”, “scared”, “afraid”, “nervous”, “jittery”, “upset”; *α* = 0.707).

In the imaging session, a 9-item visual analogue scale (VAS) (83) assessed state affect; five items tapped negative affect. For each item, donors indicated how they felt at the given moment (baseline) or during cue exposure using a 10 cm VAS with anchors of 0 (not at all) and 10 (extremely). VAS mood scores were collected at baseline and after each experimental cue. Items were projected one-by-one on the screen; a mouse designed to minimise movement was installed in the scanner. Donors' state negative affect in the baseline and trauma cue conditions was calculated by summing the five negative affect items [“anxious”, “upset”, “irritable”, “down”, and “uncomfortable”; *α* = .619, acceptable (*α* > .600) for a short scale (84)]. No anxiety composite was available for the imaging session given too few VAS items pertaining to anxiety.

To assess for any putative influence of craving-related VOCs impacting dogs' performance, donors' cannabis craving during the baseline and trauma cue was evaluated using the well-validated Marijuana Craving Questionnaire-Short Form (MCQ-SF) (85).

### 3.5 Dog training procedures

Dog training/testing took place in the CO Lab. Dogs were trained and handled by LK under the supervision of SG (qualifications in Supplementary Material S1). During training, dogs visited the lab once/week for 2-hour shifts. The training/testing protocols strictly followed positive reinforcement training methods throughout: dogs' correct behaviours were rewarded by praise and a food reward; undesired behaviours were ignored. Dogs performed an average of 3–5 sessions/day (each session = ten trials); fewer or more sessions were performed depending on the dog's motivation. Each session lasted ∼5 min, followed by a 5–20-min break, when the dog rested, played with staff, and/or was taken for a walk. When the dog expressed clear dissent [i.e., unwillingness to cooperate with the handler, expressed by the dog's attempts to remove themselves from the testing situation (86)], the work was stopped for the day and the dog rested or was taken to the park for a longer walk until their guardian retrieved them. If the dog expressed sustained dissent (86) over a longer period (e.g., a month) despite positive reinforcement principles used to enhance the dog's motivation (Supplementary Material S1), they were dismissed.

Details of the early scent-discrimination training of all the 25 dogs by following a Low Saliency Training (LST) protocol (87) and bridging the gap between LST and human breath samples are described in Supplementary Material S1. Four dogs successfully completed the LST and bridging phases and proceeded with the training for Experiment 1.

#### 3.5.1 Training for Experiment 1

**Figure 2:**
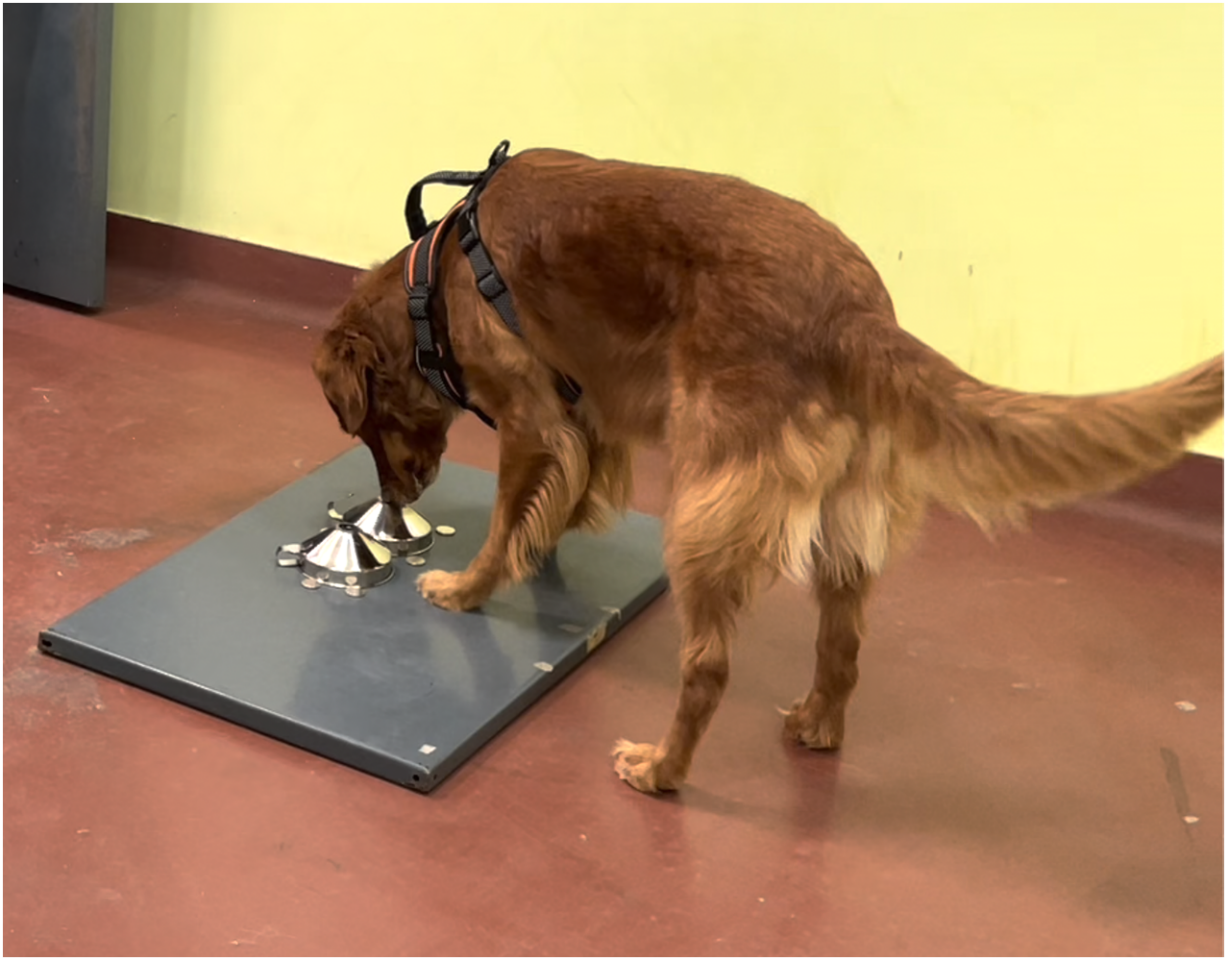
Ivy signalling the S+ in the 2AFC task.

Experiment 1 training was conducted in a two alternative forced choice (2AFC) task, where the dog was presented with a line-up of two odours: the positive stimulus (S+) and the negative stimulus (S−). The dog had to find and signal the S+ by holding their nose for 5 s on the respective container (Figure 2).

**Figure 3:**
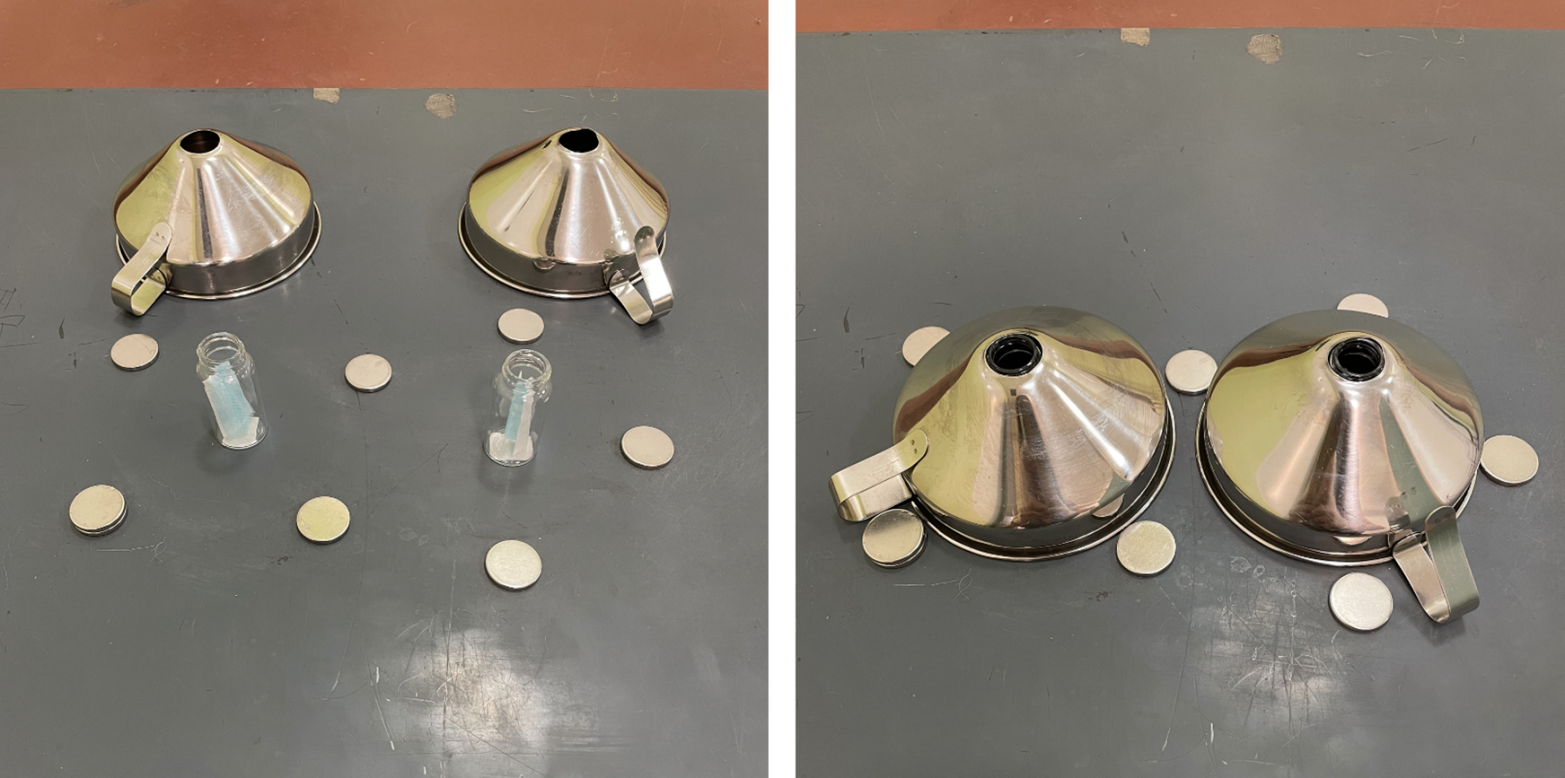
The glass vials containing samples next to the metal funnels (left), and the glass vials placed underneath the funnels as the experiment set-up (right).

Human breath samples used for training were prepared in the manner described earlier (Section 3.3). When presented to the dog, samples were uncapped and placed under stainless-steel funnels (12 cm mouth diameter, 6 cm maximum height). The funnel pipes were removed, leaving an opening on top. Funnels were attached to a metal board with magnets to prevent sliding if dogs nudged them (Figure 3). Odour placement was randomised for each trial by rolling a die (Supplementary Material S1). Only the vials containing the odours changed placement; funnels remained at the same place throughout the session.

During between-session breaks, samples were capped to preserve the odour volatiles. Each set was used for 3–5 sessions (on a single day) with one dog only. Funnels were cleaned of dog saliva with a single dry paper towel between trials. To avoid olfactory confounding, both funnels were cleaned using the same paper towel even when only one had saliva on it. After each session, funnels were cleaned with a single paper towel infused with 70% isopropylic alcohol solution and let dry completely before the next session. After the dog finished working for the day, funnels were washed with clean water and disinfected with the isopropylic alcohol solution. The metal board holding the funnels was also disinfected.

To aid the dog in learning the target odour, the handler guided the dog to sniff both samples and then signal the S+ (only before the first session). Once the dog began to exhibit signs of identifying the target odour independently (usually after 1–3 mock trials), a session was commenced. To minimise handler influence, we used a double-blind protocol. The handler and the dog waited behind a closed door between each trial, while the assistant experimenter changed (or went through the motions of changing) the odour locations according to a predetermined randomised placement order. When the trial was set up, the assistant went behind a visual barrier and called, “Ready!” The dog handler then opened the door and directed the dog to sniff the samples by saying, “Go find”. The assistant did not see the dog and the handler did not know the placement of the target odour. When the dog made a choice, the handler called the position signalled (left or right). If the dog made the correct choice, the assistant experimenter pressed the clicker, and the handler gave the dog verbal praise and a treat. If the dog made an incorrect choice, the assistant experimenter said “Thank you” in a neutral but friendly voice. This functioned as a non-reward marker to signal the trial was over and the dog could stop signalling. Without giving a reward, and in a friendly manner, the handler then called the dog behind the door to await the next trial.

During training, error correction was sometimes allowed when the dog signalled an incorrect sample, by allowing the dog to figure out the correct answer themselves or by guiding them to it. The dog received a reward after an error correction, but the trial was recorded as incorrect. As the difficulty level was very high in training, error corrections were to reduce the dog's frustration/confusion and maintain motivation (88). Error corrections were only used as much as necessary and as little as possible to prevent the dog from developing an alternative strategy of signalling different samples until hearing the click. Error corrections were not used during testing.

Dogs were considered trained and ready for testing once they began to regularly meet the passing-level criteria (80% accuracy in three consecutive sessions or 90% accuracy in two consecutive sessions) with novel donor samples and double-blind conditions. Only Ivy and Callie successfully completed this training phase. Ivy's training was complete with 12/40 sample sets (eight interview session, four imaging session) in 13 days over four months. Callie's training was complete with 16/40 sample sets (ten interview session, six imaging session) in 17 days over five months. The training period was long due to slow sample collection.

#### 3.5.2 Training for Experiment 2

**Figure 4:**
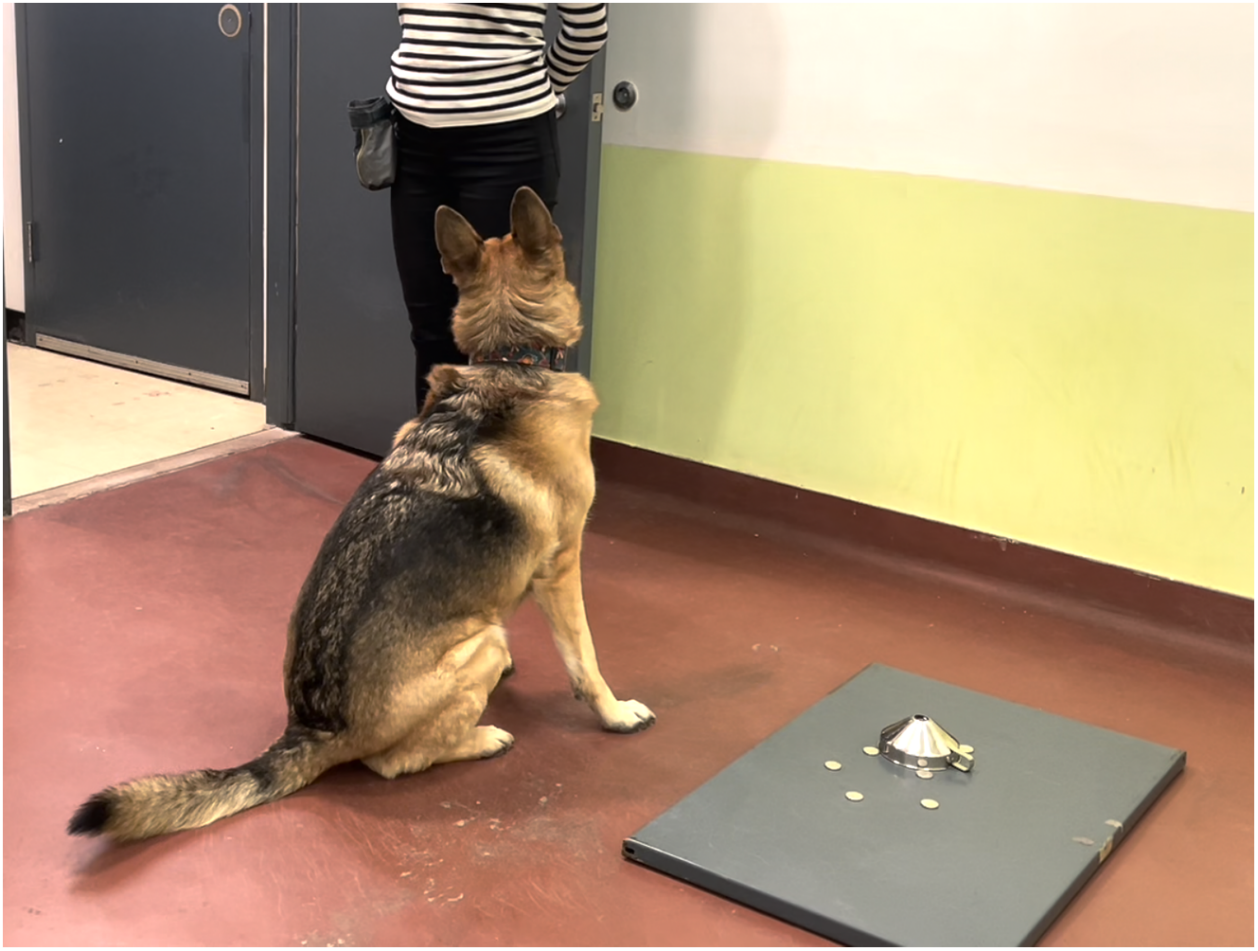
Callie signalling the S− in experiment 2 (handler in the background).

Training for Experiment 2 (yes/no detection) commenced after Experiment 1 was completed; only Ivy and Callie were trained for this task. At this point, both dogs were visiting the CO Lab 2–3 days/week. In Experiment 2, we presented one sample at a time and asked dogs to signal whether the presented sample was S+ or S− (Figure 4). The equipment remained the same; only one funnel was used. In each session, different donors' samples were presented in random order determined by a die roll.

The signal for S+ (i.e., “yes”) remained the same: a 5-s nose-hold on the funnel covering the target odour. To signal S− (i.e., “no”), we trained dogs to sit next to the sample (Figure 4). Both answers (hits and correct rejections) were equally rewarded. The double-blind procedure was identical to Experiment 1, with one exception. Instead of telling the assistant experimenter whether the dog signalled the left or right sample, the handler called out “One” or “Zero” (for “yes” or “no” dog signalling, respectively). In case of hits and correct rejections, the assistant pressed the clicker, and the handler rewarded the dog with praise/treats. For misses and false alarms, the assistant said, “Thank you” and the handler called the dog behind the door.

At first, sessions were not double-blind. The handler guided the dog towards the correct answer if the dog appeared confused; error corrections were used more liberally. To make the task easier for the dogs in the beginning, only one donor's sample set was used over the whole session and the vial containing the S− was placed on the side, while the S+ vial was always presented upright. This difference was not visible to the dogs (the samples were covered by the funnel) but placing the S− vial on the side lowered its saliency. Once the dog was able to meet the passing-level criteria for such sessions in double-blind conditions, both the S+ and S− vials were placed upright. When the dog also passed that level in double-blind conditions for at least one sample set, the next step involved sessions with multiple individuals' sets. One session always had an equal number of S− and S+ samples. Dogs were considered ready for testing when their performance was above chance in double-blind conditions. Ivy was trained for the detection task using seven sample sets (two presented only in multiple-sample-set sessions), and Callie was trained on 11 sets (seven presented only in multiple-sample-set sessions).

### 3.6 Dog testing procedures

The testing equipment was identical to the training equipment. The testing always followed rigorous double-blind methods and the sample position/condition was always randomised. All samples presented to dogs were newly prepared–mask pieces and vials used during the training were not re-used for testing. Thus, although the chemical composition of some of the samples was the same as the dogs had encountered during training (see Sections 3.6.1, 3.6.2 for details), the odorous material and the carriers were new to the dogs throughout both experiments. The S− and S+ samples continued to be prepared and handled identically within both experiments (prepared by the same experimenter and handled by the same assistant during the canine testing sessions) using the methods described above.

#### 3.6.1 Experiment 1–2AFC discrimination task

Experiment 1 targets the first hypothesis. In a 2AFC task, we tested whether dogs could discriminate between breath samples collected from the same donor during the trauma cue and baseline conditions. During this experiment, both dogs were tested on all 40 sample sets. To get sufficient data to adequately evaluate their olfactory discrimination abilities for every individual set, each dog performed three to five sessions (i.e., 30–50 trials) per set. The samples were collected over the course of 10 months. The testing was determined by the pace of sample arrival. After the initial months of training, the testing period for Experiment 1 lasted six months for Ivy and five months for Callie. As Ivy was trained using 12 sample sets, the rest of the 28 sets were completely novel to her during testing. For Callie, 24 sets were completely novel during testing.

All the samples used for training and most of the samples used for testing in Experiment 1 were presented to the dogs either on the same day or within three days of collection. However, the samples used in training (i.e., samples collected first) were presented for testing last–not knowing their expiration date, new samples were prioritised over the old ones during the testing phase. Some of these last-tested samples were thus up to eight months old. During the testing phase of the study, the dogs visited the CO Lab 2–3 times a week for a 2 h workday. Both dogs had occasional days where they did not feel like working which often presented in the dogs' poor performance. To rule out the possibility that poor performance on any particular sample set was caused by a “bad day” or sample contamination instead of the samples' olfactory properties, all sets with which the dog exhibited inconsistency (sessions with very high accuracy followed by sessions with poor accuracy) and low motivation to work (i.e., expressing dissent^3^) were re-tested on another day, where new pieces of the samples were prepared for re-testing.

The testing procedures for this task were identical to the double-blind training phase procedures described in Section 3.5.1, except for the following nuances. The first was the implementation of a warm-up trial. Although a cue sample was not prepared (to preserve the masks), it was considered important to run one to two warm-up trials before the actual testing began. The reasoning behind the warm-up trial derived from the vast variety in the VOCs of stress responses in trauma survivors. The warm-up trial before the testing thus functioned as a cue to the dog. The warm-up trial was not double-blind and the handler was ready to guide the dog to the target odour (or allow error correction) should there have been hesitation, although it was usually not necessary. To avoid confounding, it was ensured that the dog sniffed both samples during this trial. The warm-up trial was conducted only before the first session of the day and its results were not recorded. After the warm-up, the double-blind testing began.

The second nuance different from the training procedures involved recording the dogs' sample sniffing order during each testing trial as a double measure to ensure that they regularly checked both samples (although checking both samples was not a requirement to complete a trial) and to avoid sample contamination caused by one sample getting more exposure to the dog's breath than the other. In case saliva was spotted in a vial, both samples were discarded, and a whole new set of the same donor's masks was prepared to continue testing. To minimise handler influence, it was opted to not guide the dogs into checking both samples. The dogs were allowed to re-check samples before committing to an answer.

#### 3.6.2 Experiment 2–yes/no detection task

Experiment 2 targets the second hypothesis–in the yes/no task, we tested the dogs' ability to generalise, i.e., whether they could tell apart the trauma cue and baseline breath samples across different individuals when presented with one sample at a time. Furthermore, it was a confirmatory test of the dogs' sensitivity (detectability of the putative volatiles, as opposed to discriminability) with the same sample set. Each dog was tested on 21/40 sample sets in Experiment 2. As there was no objective measure to confirm the existence of stress volatiles in the trauma cue mask, the sets used in this experiment had to be confirmed as differentiable by the dogs in Experiment 1. Of all the differentiable sets, 26 with the highest performance accuracy were selected. Of these 26 sets, each dog was presented with 21 sets: 16 were common to both Callie and Ivy and five were selected individually. The reason why Experiment 2 did not involve all 40 sample sets pertains to dog welfare. By Experiment 2, Ivy began expressing sustained dissent to participating in further testing (even though she had enjoyed working throughout all the prior phases of this study). To respect her sustained dissent, it was decided to cease testing with her after roughly half of the samples had been tested. To keep the conditions equivalent for the dogs (and allow for adequate comparison of their results), Callie was tested on the same number of samples.

The testing procedures remained identical to the double-blind training procedures (all samples placed upright). No cue was used. Testing for Experiment 2 lasted two days for Ivy and one day for Callie. The testing was conducted in four sessions. As there were 21 sample sets, three sessions included five sets (five S+ and five S− samples) and one session included six sets (six S+ and six S− samples). Each sample was presented once during the session. Thus, three sessions required 10 trials and one session required 12 trials. By the time of Experiment 2, the dogs had already encountered the masks of all 40 sample sets in the previous experiment but, again, new pieces of these masks were prepared for the yes/no task for each dog. In Experiment 2, the sample age varied from 2.5 weeks to 10 months. The last encounter with these samples in Experiment 1 varied from two weeks to seven months, whereas among the samples not used during the training for Experiment 2, the longest time since the last encounter was six months.

### 3.7 Data analysis

Experiment 1 results were analysed by calculating each dog's overall accuracy and conducting binomial tests. Overall accuracy is the percentage of correct responses throughout all trials with all donor samples. The binomial test was based on the number of successful trials within the total number of trials, while the probability of success in each trial was 0.5, and the alpha-level, 0.05.

Experiment 2 gave additional information. Besides calculating the dogs' accuracy and conducting binomial tests, the yes/no detection task design enabled implementing Signal Detection Theory (SDT) (89). In contrast to a 2AFC [or any multiple alternative forced choice (mAFC)] task design, in a detection task (where odours are presented one by one), hits, misses, false alarms, and correct rejections are clearly discriminable. This enabled calculating dogs' sensitivity, specificity, and precision, as well as their response bias C and the sensitivity index independent of the bias or d'^4^ (89).

For hypothesis 3, dogs' accuracy across the trials of each sample set in Experiment 1 was correlated with donors' distress scores during sample collection (interview session: trauma cue PANAS negative affect, State Anxiety, and each item score; imaging session: trauma cue VAS negative affect, and each item score). Correlations were calculated both using Pearson's r and the non-parametric Spearman's rho, which has less statistical power than Pearson's but is also less sensitive to the outliers and, thus, more reliable. Both were calculated using Jamovi (version 2.3.16.0). Supplementary analyses were conducted to identify any other potential variables affecting dogs' ability to discriminate between baseline and trauma samples (e.g., PTSD symptom severity, PTSD diagnostic status; Supplementary Table S1).

## 4 Results

### 4.1 Experiment 1

In Experiment 1, both Ivy and Callie successfully discriminated between trauma and baseline breath samples of 36 sets. Both dogs had four sets where they performed at (or below) chance. For Callie, one of the non-discriminable sample sets belonged to her guardian (nevertheless, Callie is a regular companion dog, not a trained service dog). However, the guardian's samples represented a confound for Callie: firstly, Ivy's accuracy on that sample set was 100%, indicating the samples could have been discriminable; secondly, the preliminary analysis between Callie's performance and donor outcome measures revealed some inconsistencies in the case of her guardian's samples; and thirdly, Callie's poor performance contradicted the guardian's testimony about Callie's recent receptiveness (seemingly developed as a result of participating in this study) to the guardian's PTSD-related arousal at home. Thus, the guardian's samples were removed from Callie's data. The final number of sample sets in Experiment 1 was 39 for Callie and 40 for Ivy; the number of non-discriminable sets was three for Callie (two from the interview session, one from the imaging session) and four for Ivy (two from the interview session, two from the imaging session). For both dogs, the non-discriminable interview sample sets were all from different people. In case of the non-discriminable imaging samples, the dogs had been able to discriminate the same donor's interview samples. Only one non-discriminable set (from the imaging session) was common to both dogs.

Both dogs discriminated with 100% accuracy for nine sample sets (two common to both dogs). For Ivy, two out of these nine sets had been used during training (using different mask pieces/vials). For Callie, five out of the nine sets had been used during training. To rule out sample contamination or the “bad day” effect, Ivy was re-tested on seven sets (using new mask pieces/vials). Ivy had five “bad days” during the study, while two of the re-tested sets proved to be truly non-discriminable for her. Two sets had to be re-tested with Callie. Callie had only one “bad day”, while the other set turned out to be non-discriminable for her.

The dogs' performance in the 2AFC task is shown in Table 1. Both dogs performed at above 90% accuracy across the 36 sample sets that they could discriminate. Their accuracy was 89.5% even with the non-discriminable set results included. Both dogs' range of accuracy across the discriminable sets was 80%–100%. Across her four non-discriminable sets, Ivy's accuracy range was 30%–64%, while Callie's range across her three was 55%–60%. For the discriminable sample sets, there was no difference between Ivy's performance with the interview and imaging samples–she had above 93% accuracy with both. Callie's performance increased almost 4% with the imaging samples, although her performance was above 90% with the interview samples too.

**Table 1:** The dogs’ performance in the 2AFC discrimination task of Experiment 1.

Dog	No. of sample sets presented	No. of trials	No. of success	Binomial test *p*-value	Overall accuracy	Accuracy across discriminable sample sets (*N*)
Interview samples only						
Ivy	26	930	841	*p* < 0.001	90.81%	93.66% (24)
Callie	25	990	863	*p* < 0.001	88.19%	90.86% (23)
Imaging samples only						
Ivy	14	500	426	*p* < 0.001	86.98%	93.64% (12)
Callie	14	550	497	*p* < 0.001	91.86%	94.62% (13)
Interview and imaging samples combined						
Ivy	40	1,430	1,267	*p* < 0.001	89.47%	93.65% (36)
Callie	39	1,540	1,360	*p* < 0.001	89.51%	92.22% (36)
Total	79	2,970	2,627	*p* < 0.001	89.49%	92.94% (72)

Ivy performed 1,430 trials and Callie 1,540 trials in Experiment 1 (Table 1). Considering each session consisted of 10 trials, Ivy performed an average of 3.58 sessions (median 3) and Callie an average of 3.95 sessions (median 4) per sample set. Of the 36 discriminable sets, Ivy completed the first session with 100% accuracy for 20 sets and with 90% accuracy for 10 sets. Callie completed 13 first sessions with 100% accuracy and five first sessions with 90% accuracy. In other words, Ivy performed 83.33% and Callie 50% of all the first sessions with 90%–100% accuracy. Neither dog's performance correlated with human mask-wearing time (Supplementary Table S1).

### 4.2 Correlations between dog performance in Experiment 1 and donor distress measures

Overall, the trauma cue exposure via interview (interview session) increased the donors' experienced negative affect generally and State Anxiety specifically, and the brief audiovisual trauma cue exposure in the scanner (imaging session) increased their negative affect (Supplementary Material S2.1).

**Figure 5:**
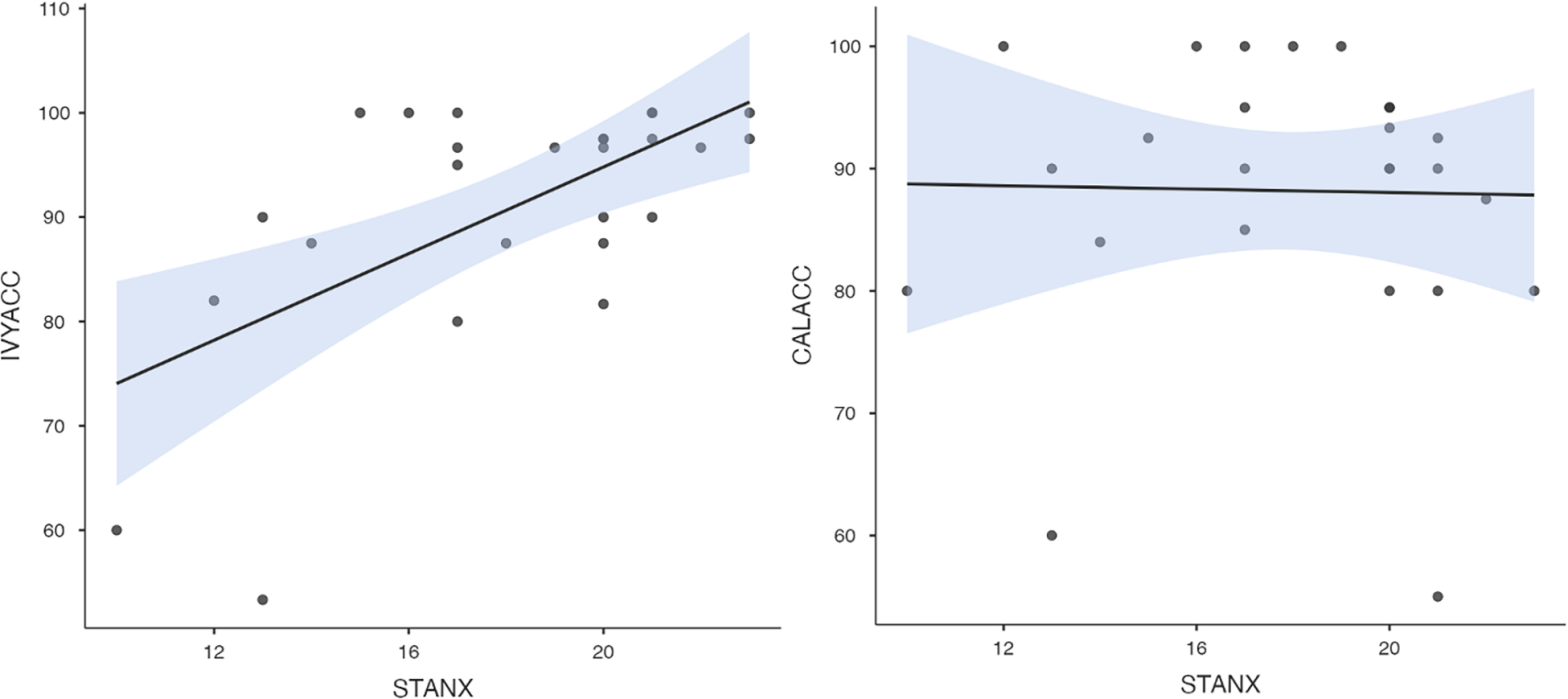
Ivy's accuracy % (left) and Callie's accuracy % (right) in the 2AFC task in relation to the donors’ self-reported State Anxiety scores during the interview session's trauma condition. The maximum value of the Y-axis is 100. The scale on the graph accommodates the standard error bar.

**Figure 6:**
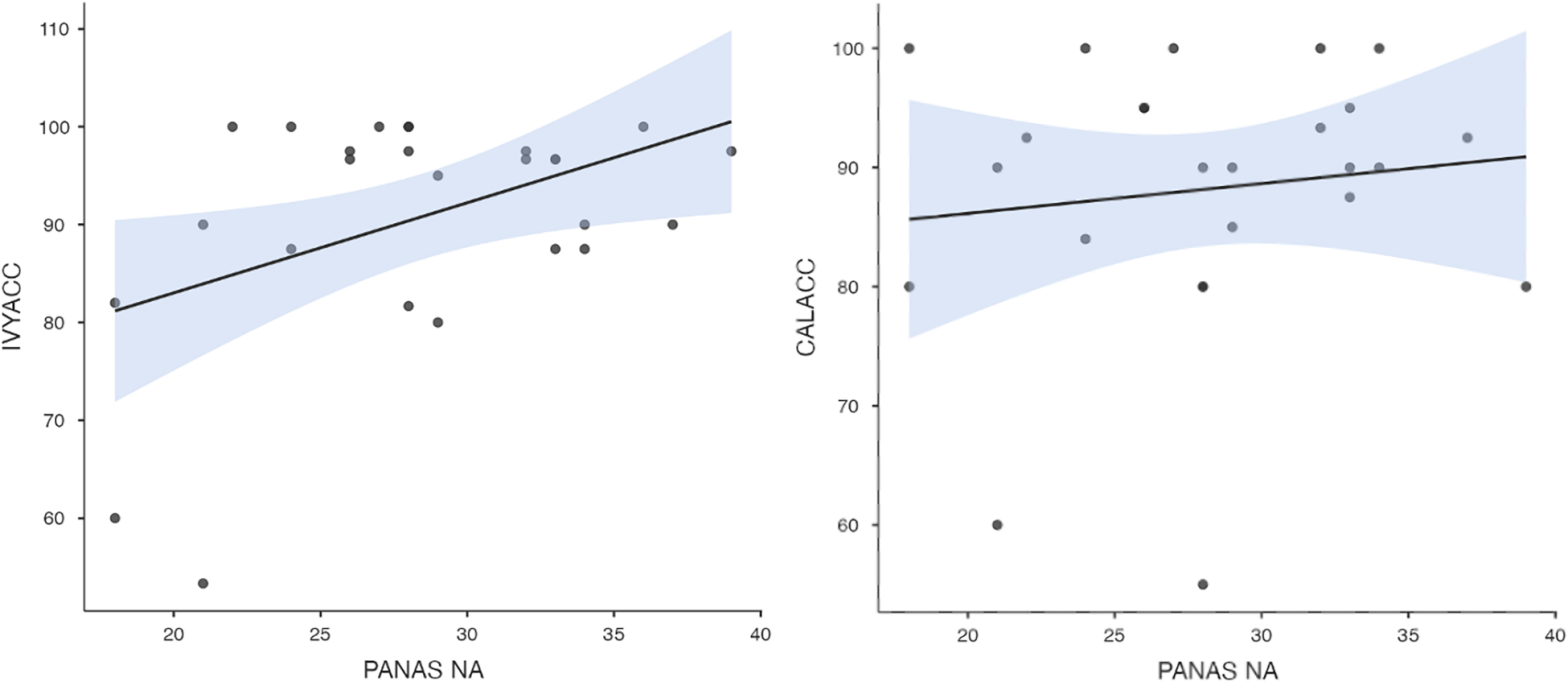
Ivy's accuracy % (left) and Callie's accuracy % (right) in the 2AFC task in relation to the donors’ self-reported PANAS negative affect scores during the interview session's trauma condition. The maximum value of the Y-axis is 100. The scale on the graph accommodates the standard error bar.

**Figure 7:**
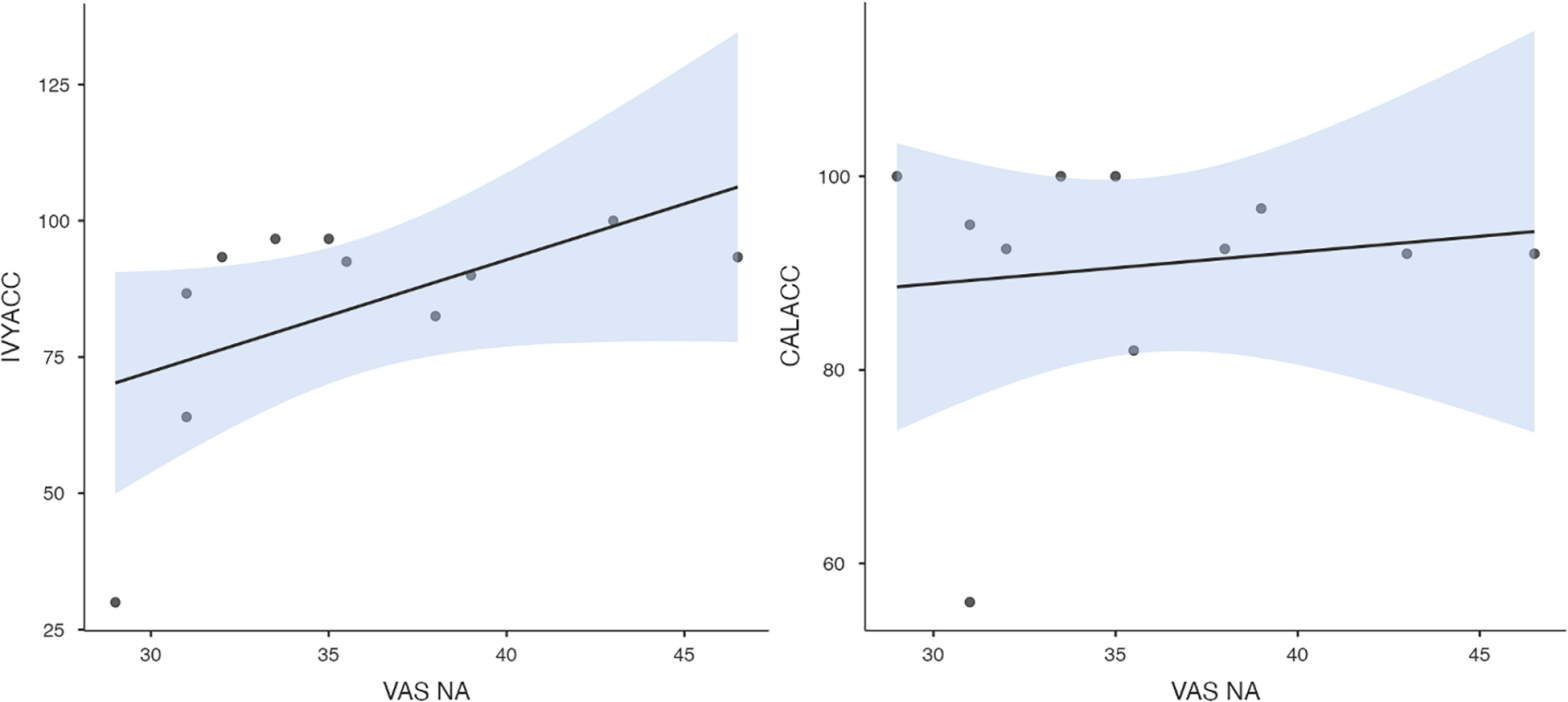
Ivy's accuracy % (left) and Callie's accuracy % (right) in the 2AFC task in relation to the donors’ self-reported VAS negative affect scores during the imaging session's trauma condition. The maximum value of the Y-axis is 100. The scale on the graph accommodates the standard error bar.

**Figure 8:**
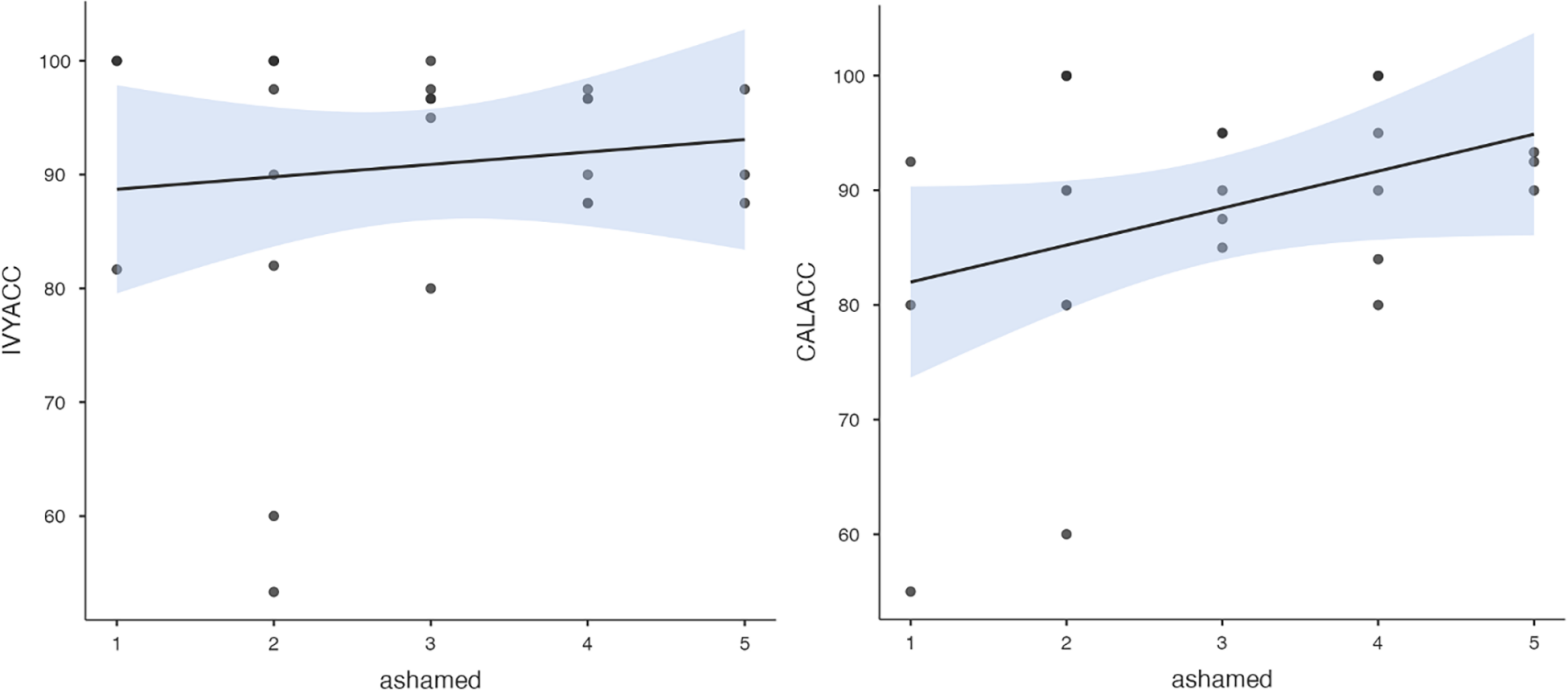
Ivy's accuracy % (left) and Callie's accuracy % (right) in the 2AFC task in relation to the donors’ self-reported feeling of shame during the interview session's trauma condition. The maximum value of the Y-axis is 100. The scale on the graph accommodates the standard error bar.

To test hypothesis 3, each dog's performance was correlated with the donors' emotional responses to the trauma cues separately for the interview and imaging sessions. Given directional predictions were made *a priori* (positive correlations expected), we used one-tailed tests (90). Using Pearson's correlations, Ivy's performance was significantly positively correlated with donors' State Anxiety (Figure 5), PANAS negative affect (Figure 6), and several PANAS negative affect items (“distressed”, “scared”, “nervous”, “jittery”, “upset”, and “hostile”), during the trauma cue in the interview session (Table 2). Ivy's performance was significantly correlated with the donors’ VAS negative affect scores after the trauma cue in the imaging session (Table 2; Figure 7). Spearman's tests confirmed these correlations in the cases of State Anxiety and the “scared” and “upset” items (interview session), and VAS negative affect (imaging session). Callie's performance was significantly positively correlated only with the trauma cue PANAS “ashamed” item in the interview session (Table 2; Figure 8). The Spearman test did not confirm that correlation.

**Table 2:** The dogs’ accuracy in the 2AFC task in relation to the donors’ self-reported negative affect (total and items) after the trauma cue.

	Ivy				Callie			
Outcome measures	Pearson's r	*p*-value	Spearman's rho	*p*-value	Pearson's r	*p*-value	Spearman's rho	*p*-value
Interview	*N* = 26				*N* = 25			
PANAS NA	0.444*	0.011	0.151	0.230	0.124	0.278	0.037	0.430
State Anxiety	0.617***	<.001	0.466**	0.008	−0.021	0.540	−0.202	0.833
Distressed	0.422*	0.016	0.320	0.055	−0.258	0.894	−0.317	0.939
Scared	0.390*	0.024	0.349*	0.040	0.287	0.082	0.114	0.294
Afraid	0.182	0.186	0.140	0.247	−0.238	0.874	−0.337	0.951
Nervous	0.376*	0.029	0.321	0.055	0.179	0.196	0.209	0.158
Jittery	0.514**	0.004	0.282	0.082	0.026	0.451	−0.073	0.635
Upset	0.548**	0.002	0.419*	0.017	−0.023	0.544	−0.028	0.553
Guilty	−0.051	0.597	−0.256	0.897	0.087	0.339	−0.026	0.549
Ashamed	0.113	0.291	−0.166	0.792	0.356*	0.040	0.263	0.102
Irritable	−0.069	0.631	−0.154	0.773	0.070	0.370	0.002	0.497
Hostile	0.337*	0.046	0.251	0.108	0.141	0.250	0.099	0.318
Imaging	*N* = 11^#^				*N* = 11^#^			
VAS NA	0.544*	0.042	0.531*	0.046	0.140	0.341	−0.282	0.799
Anxious	0.409	0.106	0.398	0.113	0.224	0.253	−0.180	0.701
Upset	−0.060	0.569	−0.025	0.530	0.454	0.080	0.187	0.291
Irritable	0.225	0.253	0.440	0.088	0.413	0.104	0.016	0.481
Down	−0.008	0.510	−0.105	0.620	−0.020	0.524	−0.108	0.624
Uncomfortable	0.453	0.081	0.266	0.214	−0.386	0.879	−0.605	0.976

* *p* < .05.

** *p* < .01.

*** *p* < .001, one-tailed.

^#^ Due to technical errors, trauma cue VAS negative affect and its items were recorded for only 11 of the 14 donors.

In supplementary analyses, we examined whether the dogs' performance was correlated with PTSD symptom severity or PTSD diagnostic measures. To evaluate whether the dogs responded to putative cannabis-related VOCs, correlations were run with donors' CUD symptom severity and cannabis craving to trauma cue exposure across the interview and imaging sessions. No significant correlations were detected (Supplementary Table S1).

Finally, a post-hoc analysis examined whether donors' PTSD symptom severity might have indirectly influenced dog performance via donors' negative emotional reactions to the trauma cues. Donors’ interview-based PTSD symptom count was significantly correlated with their trauma cue State Anxiety score (Supplementary Figure S1) which was related to Ivy's performance. Donors' self-reported PTSD symptom severity was significantly correlated with their trauma cue shame levels (Supplementary Figure S2) which was related to Callie's performance.

### 4.3 Experiment 2

The dogs' performance in the yes/no detection task is shown in Table 3. In Experiment 2, both dogs performed 42 trials (21 trauma cue, 21 baseline breath samples). As explained in Section 3.6.2, both dogs had already encountered different pieces of all these samples in Experiment 1, but up to six months prior to the testing of Experiment 2. Ivy's samples included 15 interview session sets and 6 imaging session sets collected from 16 different donors (five with samples from different stressful events, i.e., from both sessions). Callie's samples included 12 interview session and 9 imaging session sets belonging to 14 donors (six with samples from different stressful events, whereas one donor had three sample sets—two from the imaging and one from the interview session). Despite only two weeks of training for the yes/no task design, both dogs were able to generalise the target scent across different donors and the same donor across different stressful events (i.e., separate occasions of emitting stress-related VOCs) above chance.

**Table 3:** The dogs’ performance in the yes/no detection task of Experiment 2.

	Ivy	Callie
No. of samples presented	42	42
Trauma samples	21	21
Baseline samples	21	21
Correct responses	31	34
Hits	16	15
Misses	5	6
False alarms	6	2
Correct rejections	15	19
d'	1.278	1.875
C	−0.073	0.372
Accuracy	73.81%	80.95%
Sensitivity	76.19%	71.43%
Specificity	71.43%	90.48%
Precision	72.73%	88.24%
Binomial test *p*-value	*p* = 0.00097329	*p* = 0.00002684

Ivy's overall accuracy was 73.81% (range = 66.67%–90%) and Callie's was 80.95% (range = 70%–91.67%). Ivy's sensitivity and specificity were similar: she correctly identified the positive samples in 76.19% of their presentations and the negative samples in 71.43% of their presentations. Ivy's very low bias (C) suggested she is an “ideal observer”. Callie demonstrated higher specificity than sensitivity: she correctly identified the negative samples in 90.48% of their presentations, but the positive samples in 71.43% of their presentations. Her positive response bias (C) indicates a conservative decision-making strategy: when in doubt, Callie was more inclined to signal the stimulus was negative.

On no occasion was either dog able to correctly identify one person's samples collected from one session but completely unable to identify their samples from a different session. When analysing the dogs' performance with samples collected from the same donor on different stressful events, out of 20 presentations (i.e., five donors with two sets or four samples), Ivy correctly identified 3/4 samples of four donors and 4/4 samples of one donor. In Callie's case, out of 26 presentations (i.e., five donors with two sets or four samples; and one donor with three sets or six samples), she correctly identified 4/4 samples for two donors, 3/4 samples for another two donors, 2/4 samples for one donor (from different sessions), and 5/6 samples for the one donor who participated in two imaging sessions.

## 5 Discussion

Experiment 1 confirmed our first hypothesis by providing evidence that some dogs can learn to discriminate between human breath samples collected during a relatively relaxed state and during induced stress designed as an experiential analogue to a PTSD state of intrusion or hyperarousal. The dogs' performance did not depend on the length of time the donor spent wearing the mask nor on the type of experimental session (interview or imaging) from which the samples were collected. The sample collection, preparation, and handling procedures generally did not allow for any systematic differences between the baseline and trauma cue samples besides the target odour (but see Section 3.3). Combined, these data support the premise that the trauma cue-induced distress experienced by people with trauma histories manifests in changes in the person's VOC profile that some dogs can detect from breath.

Experiment 2 confirmed our second hypothesis by providing evidence that some dogs can detect the trauma cue samples from the pool of trauma cue and baseline breath samples of different individuals when presented with samples independently. Furthermore, our design where the donors underwent trauma cue exposure in two settings (interview vs. in scanner) using two cue exposure methods (interview vs. brief audiovisual cue) allowed demonstration that the dogs can perform this detection for the same individual across different stressors.

Depending on the stressor and its context, stress responses can vary in speed and magnitude of the released hormones, whether they induce adrenaline and/or noradrenaline, and which other hormones are activated (7). Different comorbid disorders can further affect the VOC profile of an individual with PTSD [e.g., an overabundance of baseline adrenaline and noradrenaline with anxiety disorders; dramatically elevated baseline glucocorticoid levels with depression (7)]. That both dogs performed with an accuracy well above chance in Experiment 2 is compatible with the premise that the stress experienced during PTSD intrusion and hyperarousal symptoms involves distinct olfactory biomarkers allowing dogs to generalise its odour across individuals and across different stressful events of one individual regardless of the complex and varied nature of the stress response associated with PTSD.

The third hypothesis of a positive correlation between the dogs' performance and the donors' self-reported negative affect during the trauma cue condition was partially confirmed. One dog's (Ivy's) ability to discriminate between the baseline and trauma cue samples in Experiment 1 was correlated with the donors' overall PANAS and VAS negative affect scores during the trauma cue conditions of the interview and imaging sessions, respectively, demonstrating Ivy's consistency in what she was detecting across the two stress-induction environments.

Both dogs' Experiment 1 performance with the interview session samples had correlations with some of the PANAS individual negative affect items. Ivy's performance showed strong positive correlations with most PANAS items reflecting humans' anxiety (i.e., distress, scared, nervous, upset, jittery), and with the State Anxiety composite, during the trauma cue exposure. Although Callie showed equally high overall accuracy as Ivy, the only human negative affect item to which she seemed sensitive was shame–one of the self-conscious emotions (91, 92). Interestingly, an emerging literature recognizes shame as a key negative emotion in trauma survivors (91–94), with some considering PTSD a “shame disorder” (95).

### 5.1 Experiment 1 implications: The olfactory biomarkers that dogs could be detecting in the stress response to trauma cue exposure

Despite the dogs' success in generalising the target odour in Experiment 2, results do not support the assumption that PTSD as a diagnosis has a disease-specific signature VOC profile as PTSD symptom count, severity, and diagnoses showed no significant relation with either dog's Experiment 1 performance. The commonality of the donors in this study was trauma exposure rather than PTSD, as 12 of the 26 donors had symptoms below the diagnostic level (CAPS-5 clinical interview). In the context of PTSD service dogs, the dogs' inability to discriminate between trauma-exposed people with and without a current PTSD diagnosis is irrelevant as the dogs would not be utilised for diagnostic purposes but, instead, as alert dogs to facilitate management of PTSD symptoms which can occur in both people with clinical PTSD and, to a lesser (but still potentially disruptive) extent, among trauma survivors with subthreshold PTSD symptoms. It is the volatiles associated with the emotional reactions to the trauma cue that the dogs should pick up on. Our results support this proposition.

However, PTSD symptoms were still a distal predictor of dog performance. For both dogs, donors' greater PTSD symptoms were indirectly associated with greater dog performance by way of donors' greater levels of specific negative affective responses to the trauma cue (State Anxiety for Ivy, shame for Callie). This suggests alert dogs may perform better with individuals with PTSD given their greater emotional responses (and presumably higher emission of VOCs) to trauma reminders.

It is often hypothesised that scent-detection dogs search for specific components or concentrations of a target odour rather than its complete signature (78, 96, 97). In Experiment 1, both dogs performed at ∼90% accuracy across all samples, yet their performance had disparate correlations with the human distress measures, indicating the dogs relied on different VOCs in the target samples. Moreover, the dogs largely differed in which sample sets they found non-discriminable suggesting they developed slightly different criteria for which VOC pattern they considered a positive sample; this phenomenon has been observed in other biomedical detection dog studies (45, 78).

Breath samples collected in the current study contain putative VOCs possibly emanating from any of the involved endocrine subsystems (Section 2.3). Although we did not measure metabolic changes in the donors, correlations between the dogs’ performance and the donors' subjective distress measures allow us to speculate on which hormones the dogs could have become conditioned. Given anxiety is robustly associated with the activation of the SAM axis and overabundance of catecholamines (7, 98), dogs could have been detecting catecholamine volatiles (adrenaline- and noradrenaline-based). This is paramount for PTSD alert dogs as the ability to perceive changes in SAM axis hormones is key to detecting early-onset stress associated with PTSD intrusion/hyperarousal symptoms. However, our study design did not permit capturing early-onset stress volatiles exclusively. The donors' minimum trauma cue mask-wearing time was 40 (interview session) to 59 min (imaging session; Supplementary Material S2.2). The HPA axis may start releasing cortisol as early as minutes or as late as hours after stressor exposure, with plasma or salivary cortisol concentrations peaking 10–30 min after stressor cessation (99). Thus, it is possible that donors started releasing cortisol while wearing the trauma cue mask. Although research has yet to explicitly demonstrate dogs' ability to detect changes in human cortisol levels, claims to this effect have been made [e.g., (100, 101)].

The strong positive correlation between Ivy's performance and the donors' State Anxiety indicates her sensitivity primarily to the SAM axis volatiles. This is consistent with a prior study (53) which provided evidence of canines' ability to detect human stress volatiles released in mere three minutes (most likely from SAM, not HPA axis). In contrast, Callie may have responded to glucocorticoid-related VOC's (e.g., from cortisol release in the HPA axis). In Experiment 1, the more ashamed the donor felt during the trauma cue, the better Callie was able to discriminate the baseline from trauma cue samples. Accumulating research suggests shame activates the HPA axis and leads to increased cortisol levels. Several studies have reported a significant positive correlation between shame and cortisol reactivity to social evaluation tasks (102–106). Moreover, a study of veterans with PTSD showed higher urinary cortisol levels in those with higher clinical shame and guilt (107). While the putative stress volatiles to which our two dogs were responding remain speculative, future research could test more directly the possibility that different alert dogs are responding to distinct volatile profiles.

### 5.2 Experiment 2 implications: Evidence for canine ability to generalise the stress associated with trauma cue exposure and additional information gained from the yes/no procedure

A number of papers have suggested using a true detection (yes/no) task (48, 87, 96). Regardless, only a few publications exist where dogs have been trained to check samples independently and give a yes/no answer using distinct signalling behaviours [e.g., (41, 108, 109)]. Experiment 2 uses this true detection procedure.

The yes/no procedure allows testing for generalisation without the possibility of the dogs relying on working memory or odour comparison. Generalisation is a result of training when the dog spontaneously learns to respond to target odour variations by learning their common properties (45, 88, 96, 110). In training biomedical detection canines, generalisation of the target odour is the goal: it enables the dog to detect the target odour in novel samples either from different people (diagnostic detection dogs) or from different medical events involving the same person (alert dogs). Experiment 2 tested generalisation ability using a true detection task: in each session, dogs were presented with samples from different donors and from separate trauma cue exposure events from recurring donors.

As we were investigating PTSD alert dog functions, ideally, we would have focused on testing dogs' ability to alert to one person's different episodes of distress/arousal with more sample sets from each donor than we had available. However, given the high interpersonal variability of human hormonal stress response, generalising the odour across individuals should be a more arduous task than generalising across different events of one individual. For example, detecting hypoglycemia in the breath samples of different people is a harder task for dogs than detecting separate hypoglycemic events in the same individual (41). Our dogs' success in detecting the trauma cue samples across individuals should thus be a good indicator of their ability to detect them across different events for the same individual.

Experiment 1 also provides evidence for generalisation: Ivy was able to successfully find the trauma cue sample in the sets of 24/26 donors and Callie in 23/25 donors. Moreover, with one exception, the dogs were able to successfully discriminate between the stress and baseline samples of the same person collected during different stressful events. Hence, the combined evidence of generalisation from both Experiment 1 and 2 endorses the possibility of training PTSD service dogs to use their acute olfaction to alert to the patient's episodes of trauma cue-related distress.

In Experiment 1, theoretically the dogs could have memorised the positive sample from each set in the first session and based their performance during the rest of the sessions on that learning–a significant confound in the field of scent-detection (45, 48). This problem can be overcome only by successful generalisation. In Experiment 2, each sample was presented just once, removing the possibility of canine performance success being the result of memory vs. olfactory skills (but see Section 5.5).

One of the main disadvantages of the mAFC procedure is dogs could learn to perform by comparing samples rather than evaluating each independently (48, 96). The yes/no procedure eliminates that risk. Although harder, the yes/no detection task has more ecological validity than the 2AFC: it better corresponds with a real-life alert task where only presence is signalled and thus provides valuable information about PTSD alert dogs' potential to detect whether the appointed person is entering an episode of distress or not.

Another advantage of the yes/no procedure is its suitability for SDT, offering information about dogs' sensory sensitivity, specificity, and response bias (89, 111). Callie's Experiment 2 results suggested a conservative response bias whereas Ivy showed no significant response bias. This information is valuable when training and selecting scent-detection dogs for operational purposes. Among biomedical detection dogs, liberal decision-makers are preferred as the consequences of giving a false alarm are much less severe than of missing a dangerous medical state (112). If Callie were to be employed as a PTSD alert dog, it might be beneficial to train her to eliminate the response bias or to opt for a more liberal bias.

Ivy's 74% and Callie's 81% overall accuracy in Experiment 2 (although lower than their 90%+ accuracy in Experiment 1) demonstrates that, despite only two weeks of training for this significantly harder task, the dogs still performed at levels comparable to the capabilities of current technology (54, 55). The dogs' performance would likely have further improved had they had more time to practise the yes/no task procedure or had all the samples been fresh (some were up to 10 months old).

### 5.3 Implications from the dogs' poor performance with certain sample sets in Experiment 1: possible reasons behind non-discriminable samples

Ivy had four and Callie three non-discriminable sets (Experiment 1). Myriad reasons independent of the samples' odour properties may explain dogs' low performance in scent-detection tasks. Both internal [e.g., medications, hydration, changes in blood flow (32, 113), stress, and diet (32, 114)] and external factors [e.g., humidity, temperature, loud noises, dog handling at work or home (114)] can impact dogs' day-to-day olfactory capabilities and motivation to work. To account for these factors, the dogs were re-tested on a different day on every set with which they exhibited lack of motivation (i.e., expressed dissent) and inconsistency, or when any other deviations from the dogs' usual working conditions were observed (e.g., suffering from obvious health issues, such as hotspots). New mask pieces were prepared on such occasions, ruling out sample cross-contamination. The underlying cause for the dogs' low performance with sets that remained non-discriminable regardless of day most likely involved the samples' biochemical properties.

First, in the case of non-discriminable samples, the dog may have found the person's scent attractive, aversive, or otherwise distracting (112) as with the guardian's breath samples presenting a confound for Callie. Second, given all donors were regular cannabis users for reasons related to the parent study (70, 79) and since cannabis abstinence was verified verbally, it is possible some nonetheless used cannabis on the test day and this scent obscured volatile differences between the trauma cue and baseline samples. Third, while we know the trauma cue exposure was effective overall in raising humans' self-reported negative affect relative to baseline, we do not know how their distress manifested endocrinologically, whether their baseline and trauma cue masks differed in stress-related VOCs, and whether different samples contained a predominance of different VOCs (e.g., adrenaline vs. cortisol) detected differentially by the two dogs.

Unfortunately, the pool of non-discriminable samples in this study was too small to provide any firm evidence to support the above-mentioned propositions. Interestingly though, in all but one case, the non-discriminable samples for one dog were discriminable for the other. Reasons why one imaging session set was non-discriminable to both dogs could vary from the donor's diet and cosmetic products to them having high anticipatory anxiety prior to entering the scanner leading to indistinguishable olfactory properties of the baseline and trauma cue masks.

### 5.4 Potential confounding biases

The methodology of canine scent-detection studies ought to be scrutinised for any systematic differences between the target and non-target odours to avoid biased or confounded results. Even though great effort was put into conducting the experiments in a way that would minimise the risks of bias or confounds, several potential sources remain. When scrutinising sample collection methods, one could argue that, in case of the imaging sets, the smell of the scanner, and the fact that the imaging session baseline and trauma cue masks were not identical (possible different manufacturers; lack of metal wire in trauma mask) could have caused confounding. Furthermore, during the imaging session, the donors' experienced stress induced by the cue exposure vs. the scanner could not be differentiated. Moreover, since donors had the choice to opt out from the imaging session (Section 3.3), those who participated in the imaging session may differ from those who did not in important ways (e.g., avoidance levels).

However, there was no indication of dogs being able to discriminate the imaging session samples significantly better/worse than interview session samples. Thus, it is plausible to assume that the dogs learned to ignore any systematic differences between the imaging session trauma cue and baseline masks and focused on the target odour instead. Any donor-specific differences and the added stress due to the brain scanning appears to not have affected the dogs' ability to discriminate between the trauma cue and baseline samples when compared to their performance with the interview samples.

Another potential methodological criticism involves the trauma cue mask also being worn during the neutral and cannabis cues. It is doubtful that the neutral cue could have presented a confound. However, if there are distinct putative VOCs emanating during cannabis craving, the cannabis cue could have presented a confound. As mentioned, the dogs’ performance was neither correlated with the donors' CUD symptom severity nor with their cannabis craving combined across sessions.

Some procedural aspects may have confounded results. Although measures were taken to avoid sample contamination with dogs' breath and saliva (Sections 3.5.1, 3.6.1), in Experiment 1, the positive sample may have gotten gradually more contaminated than the negative sample as the signal for S+ involved holding the nose on the vial for five seconds. Had the dogs learned to perform in Experiment 1 by detecting their own breath rather than the target odour, a pattern should have occurred where the performance was low in the first session and improved during the later sessions; no such pattern was observed. Indeed, the dogs performed at lower accuracy during the last sessions, possibly as they grew tired or their own accumulated breath volatiles began over-shadowing the sample odour.

Another example is the repeated use of samples within one testing day in Experiment 1. Various biomedical scent-detection studies [e.g., (32, 39, 78)] have emphasised the importance of minimising the presentations of each sample to avoid the dogs developing the alternative strategy of memorising the positive sample in each set. However, repeated presentations allow a valid assessment of dogs' discrimination accuracy for each set and of their average accuracy across sets. The dogs' generally high performance during first sessions attests to their reliance on olfaction rather than memory. Furthermore, as explained earlier, Experiment 2 established generalisation as opposed to memorisation.

Lastly, the use of self-report to measure human response to the trauma cue exposure could have minimised correlations between the dogs' performance and donors' response. Although we selected measures with good psychometric properties, and tapped subjective experience to which only the individual has access (115), self-reports entail a relatively high risk of bias including minimising or misinterpreting emotional symptoms (116–120). Thus, self-reports are informative but imperfect reflections of the VOCs emitted during trauma cue exposure. Although PANAS negative affect was significantly higher during the trauma cue vs. baseline condition across donors, 6/26 reported their negative affect during the trauma interview to be equal to or lower than at baseline. Callie was able to correctly indicate the trauma cue sample in case of all these donors' sets and Ivy in case of five. These discrepancies between donors' self-reports and dog performance suggest the intriguing possibility that dogs may detect stress more reliably than people perceive (or report) it. Testing this possibility in future would require using a validated physiological index of stress to the trauma cues as the gold standard and compare the accuracy of donor self-reports and dog performance as stress markers. If so validated, it would support training PTSD service dogs to alert to an upcoming episode of distress before the person is aware of it.

### 5.5 Limitations and future implications

Several limitations should be considered when interpreting study results. First, although the psychological distress measures proved valuable and informative, they have a relatively high risk of bias. Future studies should corroborate subjective self-report findings with more objective physiological stress measures and blinded behavioural observations to confirm each sample's status as target or non-target. A predetermined sample status would facilitate dog training, provide further insight into dogs' performance, and eliminate mistaking the dogs' correct responses as false (e.g., when the baseline is equally or more “stressed” than the experimental condition). However, the endocrinology of human stress response varies so widely that a stress sample can include the target odour or not depending on the VOCs to which the dog is sensitive. To get better insight into which stress-related VOCs dogs are detecting, future studies should measure the changes in the human donors' endocrinological profile (including SAM and HPA axis hormones) during the trauma cue vs. baseline conditions.

Another limitation is that most samples likely included both early and late stress VOCs as the donors wore the trauma mask for a relatively long duration. Results provided indirect evidence for canine olfactory ability to detect early-onset stress experienced by PTSD patients in response to trauma cue exposure: strong correlation of Ivy's performance with the donors' State Anxiety (associated with SAM axis hormones); and both dogs' abilities to detect stress VOCs in the breath of donors whose self-reported negative affect during the trauma cue was equal to or lower than at baseline. Given PTSD alert dogs must detect early-onset stress VOCs, future studies should test dogs' olfactory acuity on samples collected very shortly (mere minutes) after the trauma cue.

This study may also be criticised for using donors who were regular cannabis users. Although cannabis use was a constant across donors and conditions, it is still possible that our results only generalise to donors who are chronic cannabis users due to alterations in their endocrine stress response. However, chronic cannabis use has been associated with a blunted HPA axis response and heightened baseline cortisol (121–123). Thus, it should be more difficult for dogs to detect stress VOCs released to trauma cue exposure and to discriminate the baseline and trauma cue breath samples of cannabis users relative to non-cannabis users. The fact that the dogs demonstrated this ability even with cannabis users makes our proof-of-concept more stringent.

Further limitations include the repeated use of training samples during testing and using the discrimination task samples again in the detection task. Ideally, training samples would not be reused for testing (45, 78). Given challenges in recruiting donors into such a psychologically- and emotionally-taxing study, and collecting breath samples during the COVID-19 pandemic, obtaining enough samples to avoid reusing training samples for testing was not feasible. For Experiment 1, Ivy was trained on 12 and Callie on 13 of the total of 40 sample sets (the rest of the samples were novel to the dogs during testing). For Experiment 2, Ivy was trained on 7 and Callie on 11 of the 21 sets (but both dogs had encountered the rest of the samples in Experiment 1). While dogs have been documented to memorise up to 40 distinct odours (such as vanillin, tea tree oil, and parmesan) (124), in our study, the dogs would have had to memorise 40 target odours from the pool of 80 very similar odours with minute putative differences (40 trauma cue and 40 baseline samples). Furthermore, regular maintenance training (more often than once a month) with memorised odours is usually required to preserve dogs’ performance accuracy over time (125, 126), although recent work suggests that dogs can maintain their discrimination (but not detection) accuracy of less complex odours for up to 12 months without maintenance training (124). Our study design did not enable maintenance training: since the samples used for training were tested last in Experiment 1, the dogs' last encounter with these samples was up to eight months prior to testing. In Experiment 2, the dogs' last encounter with the samples not used for training was up to six months prior to the testing. We also reduced the risk of the dogs memorising the positive samples by preparing new mask pieces of the samples for each testing day. It was observed that the dogs' performance did not depend on the sample age in either of the Experiments. Moreover, no significant difference was observed in dog performance with novel samples vs. samples used for training. All things considered, the dogs' reliance on long-term memory appeared highly unlikely. Regardless, more research is needed using more complex stimuli (like ours) and larger number of target odours to determine the limits of canine long-term olfactory memory.

There is no concrete standard on how many unique samples are required for training scent-detection dogs for biomedical tasks (96). The number of samples used to train dogs to detect cancer has varied from 26-to-several hundred target samples and 16-to-500 non-target samples (96). Our dogs' Experiment 2 performance indicates that 40 target and non-target samples were sufficient to enable generalisation. Nevertheless, with relatively few samples, ours should be considered a proof-of-concept study. Future studies of canine olfactory learning in relation to PTSD should aim for a higher number of unique samples to ensure no repeated use and more firmly rule out memory as having contributed to our findings [a minimum of 100 target and non-target samples has been recommended for training complex tasks (96)]. The goal should be to collect as many samples as possible from the same donors to investigate how reliably dogs can alert to different episodes of trauma cue-induced stressful arousal in one person. Another future direction would be to include stress samples from a non-trauma-exposed population in the pool of testing (but not training) samples. This would potentially yield some evidence on whether the stress volatiles theoretically associated with PTSD intrusion/hyperarousal symptoms differ from those of a normal (non-clinical) stress response.

## 6 Conclusion

This study is the first to demonstrate canine olfactory ability to detect putative VOCs emitted by people with trauma histories when experiencing distress theoretically associated with PTSD intrusion/hyperarousal symptoms. These results are consistent with other studies that have provided preliminary evidence of dogs' ability to detect stress in humans by using their olfaction. We add to this evidence-base by expanding from stress to trauma (all donors reported trauma histories and more than half had PTSD) and employing well-validated trauma-related stress-induction measures. Our results do not suggest that PTSD has a disease-specific signature VOC profile. Instead, the results are aligned with the premise that the endocrinology of the acute stress response to exposure to personalised trauma cues can be detected by dogs. Moreover, the results demonstrated that dogs can generalise the olfactory biomarkers of this stress response, making the idea of PTSD alert dogs plausible.

However, the olfactory biomarkers that dogs generalise may vary, with different dogs detecting different olfactory biomarkers in human trauma cue breath samples. While both dogs performed at high accuracy in the 2AFC odour discrimination task of Experiment 1, the differences in correlation of the dogs' performance with the donors' specific distress measures indicate dogs may have been attuned to separate endocrine stress markers. Although changes in endocrine parameter levels in donors were not recorded, we hypothesise that one dog was detecting the SAM axis hormones, while the other was sensitive to the HPA axis hormones. Future studies should test this hypothesis by measuring levels of the respective hormones. As the concept of PTSD alert dogs requires detecting the early onset of stressful arousal, canine ability to detect the SAM axis hormones is particularly important to confirm.

The yes/no detection task in Experiment 2 brought the experimental conditions closer to real-world settings for PTSD alert dogs. Furthermore, Experiment 2 was the first to examine dogs’ sensitivity and specificity in detecting putative human stress VOCs. Results showed that dogs possess a comparable or higher sensitivity and specificity than current technology (e.g., solid-phase microextraction, gas chromatography-mass spectrometry, and “electronic noses”). Dogs have other advantages over technology in assisting people with PTSD as dog functions extend far beyond alerting to and distracting from upcoming distress episodes [e.g., offering unconditional love and support (24, 26)].

In conclusion, this study provides information on mechanisms underlying the alert function of PTSD service dogs, as well as evidence for the possibility of training dogs to detect upcoming distress episodes through the person's breath. This skill would enhance dogs' alert function by enabling even earlier distraction (i.e., before the onset of observable cues), potentially leading to the person's enhanced comprehension of their symptoms, more efficient application of coping skills learned in therapy, and prevention of the episode from “spiralling”. Nevertheless, as this is a proof-of-concept study, validation studies are required to confirm these promising results.

## Data Availability

The raw data supporting the conclusions of this article will be made available by the authors, without undue reservation.

## Footnotes

^1^This works the other way around as well: an aroused sympathetic nervous system stimulates the amygdala (7), resulting in a vicious circle characteristic of PTSD/anxiety disorders.

^2^Due to technical errors with the imaging equipment in one donor's first imaging session, a significant amount of parent study data (69) was lost. This donor re-did the imaging session a different day. We utilised the breath samples from both sessions, as all were adequate. This was the only donor who donated three sample sets (one from the interview, two from the imaging sessions).

^3^Examples of how the two dogs in our study expressed dissent when asked to go sniff the samples include the following behaviours: remaining in the waiting area and sitting/lying down and/or staring at the handler; going to the assistant experimenter instead; sniffing the floors/walls/doors of the experiment room instead of samples; pawing at the door leading to the rest area; initiating play; mounting the handler and thrusting; restlessness (motor); sweaty paws (leaving paw prints on the floor); whining/whimpering; yawning; lip-licking; barking. The presence of two or more such behaviours in the beginning of testing day (already during the first session) was interpreted as indicative of the dog's low motivation to work on a given day.

^4^Accuracy % = (hits + correct rejections)/(hits + misses + correct rejections + false alarms) × 100 Sensitivity % = hits/(hits + misses) × 100 Specificity % = correct rejections/(correct rejections + false alarms) × 100 Precision % = hits/(hits + false alarms) × 100 C = –(Z_hits_ + Z_false alarms_)/2 d’ = Z_hits_ – Z_false alarms_ The *z* score represents standard deviation units.
