# Supplementary material for: Can scent-detection dogs detect the stress associated with trauma cue exposure in people with trauma histories? A proof-of-concept study
Source: Front Allergy. 2024 Mar 28;5:1352840. doi: 10.3389/falgy.2024.1352840 (PMC11006987; doi:10.3389/falgy.2024.1352840)
Supplement: Supplementary file 1 [file Datasheet1.pdf]

Supplementary Material

1 Supplementary information on dog training procedures

Dog training was carried out by the first author, who, prior to the study, had five years of positive reinforcement training experience with captive wild, as well as domesticated animals. The training process was supervised by the director of the Dalhousie CO Lab, Dr. Simon Gadbois, who has decades (1990-present) of experience in training scent-processing dogs, as well as a background in animal learning and animal olfactory psychophysics.

The first phase of the training involved introducing the dog to the clicker (if the dog was naïve to it), shaping the signalling of the positive stimulus (S+), and encouraging sniffing by introducing the negative stimulus (S-). S+ at that phase was a regular cotton ball infused with 4 mL of a 15-minute brew of Orange Pekoe tea and S- was a regular cotton ball infused with 4 mL of water of equal temperature.

The second phase followed a Low Saliency Training (LST) protocol. LST was carried out in the form of a 2AFC task. In this phase, the dog was trained to search for Orange Pekoe tea of gradually decreasing saliency, starting from a 15-minute brew and ending with a 1:3 dilution of a 5-second brew and water. The S- was water of equal temperature. The cotton balls infused with tea were inserted in 20 mL glass vials, the glass vials were placed under stainless steel funnels without a pipe (24 cm mouth diameter, 11 cm maximum height). The funnels were attached to a metal board with magnets.

The placement of the odours was randomised for each trial by rolling a die. To avoid the dog developing a side bias, both S+ and S- had to be on each placement (left or right) for an equal or close to an equal number of trials in each session (five or six occurrences on one side with five or four, respectively, on the other side were accepted), and the same placement was not allowed for more than three trials in a row. To prevent the dog from developing an alternative strategy, close attention was paid to avoid any obvious placement patterns. To pass to the next level of LST (with more decreased saliency), the dog had to complete three sessions in a row with at least 80% accuracy or two sessions in a row with at least 90% accuracy. Each session consisted of ten trials. All the trials during LST were double-blind.

To bridge the gap between LST and human breath samples, tea breath had formerly been used in Dalhousie's CO Lab (1). In this scenario, S+ was an Orange Pekoe tea breath sample collected by having a person slosh tea in their mouth for 30 seconds before spitting it out and breathing on a silicone-coated cotton ball (2) through a breath collection tube. S- was a blank silicone-coated cotton ball. Subsequently, S+ and S- were breath samples of different people and, as a third transitioning step, S+ and S- were breath samples of the same person collected at different times of day. Although, at first, we tried to follow the same protocol and the dogs passed the tea breath level with flying colours, we soon noticed that the clean breath levels were counterproductive as they were predisposing the dogs to detect human individual VOC profiles or focus on irrelevant aspects of human breath. To prevent the dogs from having any conditioned bias before introducing them to the human participant breath samples, we decided to omit the breath-training phase.

As the 1:3 dilution of a 5-second brew and water on regular cotton balls appeared more challenging to the dogs than tea breath on silicon-coated cotton balls, we returned to the weakest-saliency liquid tea levels and increased the task difficulty by introducing new target odours and a competing non-target odour. On each day, the dog had a different target odour to find. S+ was a cotton ball with a new tea and S- was another tea of equal saliency and temperature (Orange Pekoe was not used from then on). The training procedures remained the same, apart from the addition of a cue, thus making it a 2AFC matching-to-sample task.

Namely, before asking the dog to go sniff the samples, the handler presented the dog with a cue (i.e., another glass vial containing a cotton ball with the S+), to communicate to the dog which odour to search for. This phase was designed to prepare the dogs for human participant samples by weaning them off the Orange Pekoe and by adding distractions (equally salient non-target odours) that would encourage paying attention to minute differences of similar scents. The LST and bridging phase were completed in approximately six months but was continued to maintain the dog's performance until receiving participant samples.

At any point during the study, if the dog expressed sustained dissent (3) over a longer period (e.g., a month) and no amount of rest, walks, olfaction-based games, or upgrade of the reward value (including mixing the rewarding value of treats in a treat bag in an attempt to utilise the anticipatory contrast effect to enhance the dog's motivation; (4–6)) succeeded in increasing the dog's willingness to work, they were dismissed from the program. In the field of bioethics, sustained dissent is typically used to discuss human research participants, but it is increasingly discussed in animal bioethics. In the current study, sustained dissent is viewed as the animal's continued unwillingness to cooperate with the handlers/researchers, expressed by the animal's attempts to remove themselves from the testing situation (3).

2 Supplementary results

2.1 Supplementary manipulation check of PANAS and VAS negative affect across trauma cue vs. baseline conditions

The human donors' mean PANAS negative affect score at baseline in the interview session was 22.85 (median=21, possible range=10–50, actual range=11–41). The donors' mean PANAS negative affect score during the trauma cue in the interview session was 28.46 (median 28, actual range 18–39), a statistically significant increase from baseline ($p<.001$). The donors' mean State Anxiety score at baseline in the interview session was 14.15 (median=14, possible range=6–30; actual range=6–29). During the trauma cue in the interview session, it was 18.08 (median=19.50, actual range=10–23), a statistically significant increase from baseline ($p<.001$).

Donors' mean VAS negative affect score at baseline in the imaging session was 17 (median=20.50, possible range=5–50, actual range=5–27.50). Their mean VAS negative affect score during the trauma cue was 35.77 (median=35, actual range 29–46.50), a statistically-significant increase from baseline ($p=0.001$). Due to technical and human errors, VAS negative affect was recorded for only 11 of the 14 donors after the trauma cue and 7 donors after the baseline condition.

2.2 Supplementary information on the human donors' mask-wearing time

The average time of wearing the baseline mask during the interview session was 29.35 min (median=31 min, range=15 min–48 min). The average time of wearing the trauma mask during the interview session was 78.96 min (median=75 min, range=40 min–120 min). The average difference of time wearing the baseline mask and time wearing the trauma mask in the interview session was 49.62 min (median=45, range=17 min–105 min) with trauma masks always being worn longer. The average time of wearing the baseline mask during the imaging session was 17.14 min (median=15 min, range=5 min–32 min). The average time of wearing the trauma mask during the imaging session was 69.57 min (median 70 min, range 59 min–102 min). The average difference of time wearing the baseline mask and time wearing the trauma mask in the imaging session was 52.43 min (median=55 min, range=35 min–70 min) with trauma masks always being worn longer.

3 Supplementary Figures and Tables

3.1 Supplementary Figures

Supplementary Figure 1. The correlation between the human donors' PTSD total CAPS-5 symptom count (left) or PCL-5 symptom severity (right) and self-reported State Anxiety PANAS composite during the interview session's trauma condition.

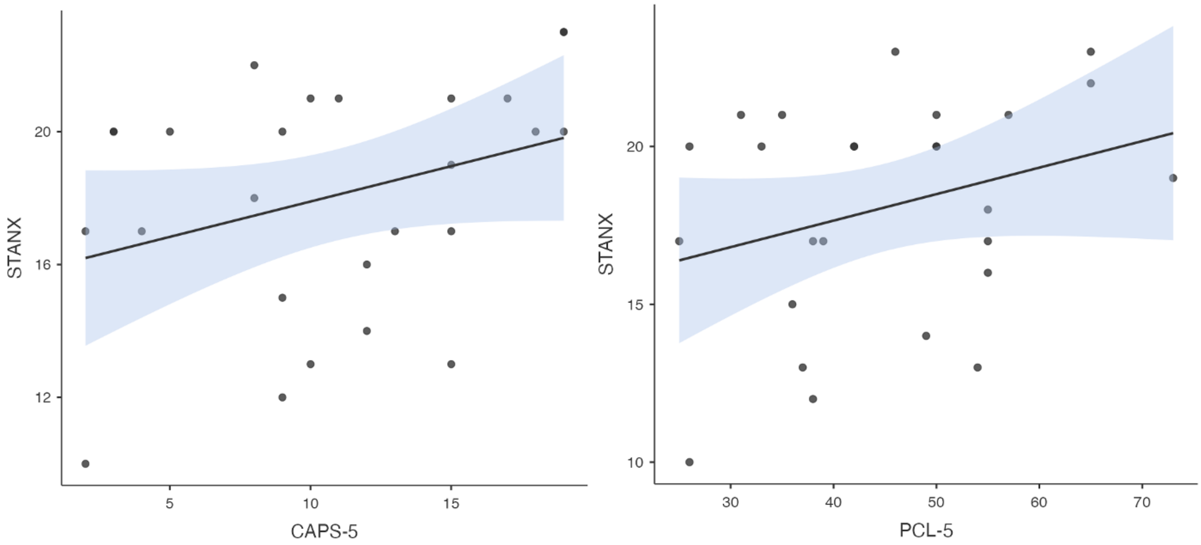

Note. The scale of the Y-axis is 6–30. The scale of the X-axis is 0–20 (left) or 0–80 (right).
In case of CAPS-5: Pearson's r=0.332, p-value=0.049; Spearman's rho=0.334*, p-value=0.048.*
In case of PCL-5: Pearson's r=0.304, p-value=0.066; Spearman's rho=0.261, p-value=0.099.
*N=26; one-tailed; * indicates significant correlations.*

Supplementary Figure 2. The correlation between the human donors' PTSD total CAPS-5 symptom count (left) or PCL-5 symptom severity (right) and self-reported shame response on the PANAS during the interview session's trauma condition.

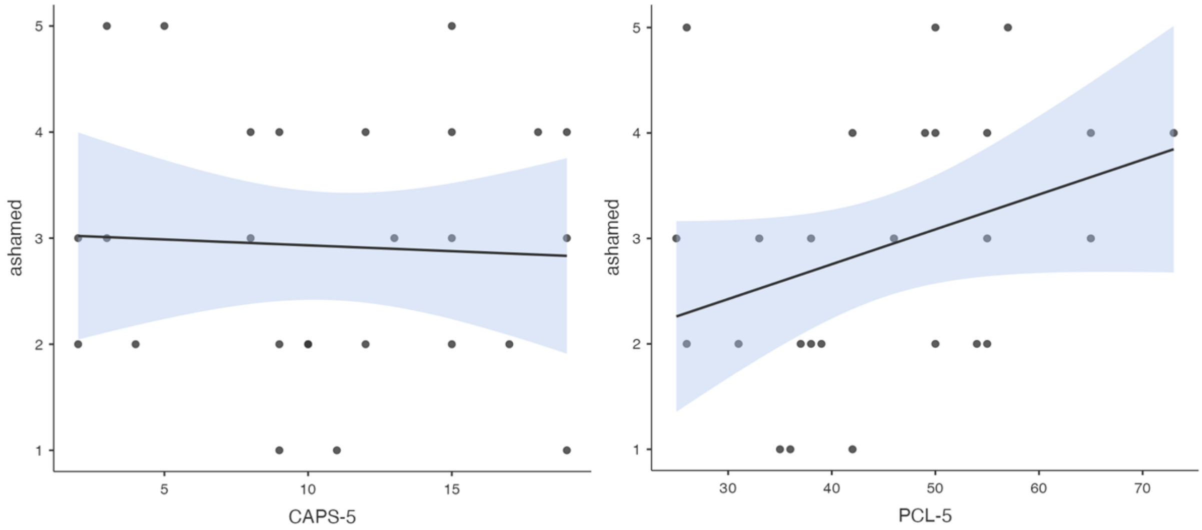

Note. The scale of the Y-axis is 1–5. The scale of the X-axis is 0–20 (left) or 0–80 (right).
In case of CAPS-5: Pearson's r=-0.049, p-value=0.594; Spearman's rho=-0.031, p-value=0.559.
In case of PCL-5: Pearson's r=0.342, p-value=0.044; Spearman's rho=0.374*, p-value=0.030.*
*N=26; one-tailed; * indicates significant correlations.*

3.2 Supplementary Tables

Supplementary Table 1. The dogs' accuracy in the 2AFC task in relation to the donors' PTSD and CUD severity, cannabis craving score, and mask-wearing time.

	IVY				CALLIE			
**Outcome measures**	**Pearson's r**	**p-value**	**Spearman's rho**	**p-value**	**Pearson's r**	**p-value**	**Spearman's rho**	**p-value**
**COMBINED**	N = 40				N = 39			
MCQ-SF trauma cue¶	0.212	0.104	0.197	0.121	0.230	0.089	0.164	0.170
Mask-wearing time§	0.044	0.393	-0.022	0.553	-0.078	0.681	-0.059	0.640
**INTERVIEW**	N = 26				N = 25			
PCL-5#	0.000	0.500	-0.175	0.804	0.229	0.136	0.182	0.192
CAPS-5#	0.016	0.469	0.001	0.498	-0.125	0.724	-0.203	0.835
CUDIT-R#	-0.281	0.918	-0.241	0.882	-0.084	0.656	-0.179	0.804
Mask-wearing time§	0.180	0.190	0.079	0.350	-0.048	0.591	-0.039	0.574
**IMAGING**	N = 14				N = 14			
Mask-wearing time§	-0.170	0.720	-0.167	0.716	-0.217	0.772	-0.049	0.567

** p < .05, ** p < .01, *** p < .001, one-tailed.*

¶ N=37 for Ivy and N=36 for Callie as, due to technical errors, MCQ-SF scores were not recorded in case of three donors during the imaging session.

§ Mask-wearing time refers to the difference of the time during which the baseline and trauma cue masks were worn by the donor.

# The correlations were run only with the interview session samples. The imaging samples were left out to avoid using the scores of some human participants twice.

Supplementary references

1. Gadbois S, Reeve C. The semiotic canine: Scent processing dogs as research assistants in biomedical and environmental research. Dog Behav. 2016;2(3):26–32.
2. Reeve C, Wentzell P, Wielens B, Jones C, Stehouwer K, Gadbois S. Assessing individual performance and maintaining breath sample integrity in biomedical detection dogs. Behav Processes. 2018;155:8–18.
3. Fenton A. Holding animal-based research to our highest ethical standards: Re-seeing two emergent laboratory practices and the ethical significance of research animal dissent. ILAR J. 2020;60(3):397–403.
4. Bremhorst A, Bütler S, Würbel H, Riemer S. Incentive motivation in pet dogs – preference for constant vs varied food rewards. Sci Rep. 2018;8(1):9756.
5. Flaherty CF. Incentive Relativity. New York, NY, US: Cambridge University Press. 1996.
6. Riemer S, Thompson H, Burman OHP. Behavioural responses to unexpected changes in reward quality. Sci Rep. 2018;8(1):16652.
